# Safety of sodium-glucose transporter 2 (SGLT-2) inhibitors in patients with type 2 diabetes: a meta-analysis of cohort studies

**DOI:** 10.3389/fphar.2023.1275060

**Published:** 2023-10-13

**Authors:** Chun Xing Li, Tian Tian Liu, Qian Zhang, Qing Xie, Xu Hua Geng, Chun Xia Man, Jia Yi Li, Xin Ying Mao, Yue Qiao, Hua Liu

**Affiliations:** ^1^ Department of Pharmacy, Aerospace Center Hospital, Beijing, China; ^2^ College of Pharmacy, Hebei Medical University, Shijiazhuang, China; ^3^ Aerospace School of Clinical Medicine, Peking University, Beijing, China

**Keywords:** sodium-glucose transporter 2 inhibitors, safety, diabetic ketoacidosis, lower limb amputation, urinary tract infections, genital tract infections, meta-analysis

## Abstract

**Aims:** This study aimed to investigate the association between the use of sodium-glucose transporter 2 inhibitors (SGLT-2i) and the risk of diabetic ketoacidosis (DKA), lower limb amputation (LLA), urinary tract infections (UTI), genital tract infections (GTI), bone fracture, and hypoglycemia in cohort studies.

**Methods:** A systematic search was conducted in the PubMed and Embase databases to identify cohort studies comparing the safety of SGLT-2i versus other glucose-lowering drugs (oGLD) in patients with type 2 diabetes mellitus (T2DM). The quality of the studies was assessed using the Newcastle-Ottawa Scale. Primary endpoints were DKA and LLA, while secondary endpoints included UTI, GTI, bone fracture, and hypoglycemia. Hazard ratios (HR) with 95% confidence intervals (CI) were calculated.

**Results:** A total of 9,911,454 patients from 40 cohort studies were included in the analysis. SGLT-2i use was associated with a higher risk of DKA (HR: 1.21, 95% CI: 1.07–1.38, *p* = 0.003) and GTI (HR: 2.72, 95% CI: 2.48–2.98, *p* < 0.01). However, it was not associated with an increased risk of LLA (HR: 1.06, 95% CI: 0.92–1.23, *p* = 0.42), UTI (HR: 0.99, 95% CI: 0.89–1.10, *p* = 0.83), or bone fracture (HR: 0.99, 95% CI: 0.94–1.04, *p* = 0.66). Furthermore, SGLT-2i was associated with a reduced risk of hypoglycemia. Furthermore, compared to dipeptidyl peptidase 4 inhibitors, SGLT-2i as a class and individually was associated with an increased risk of DKA. Canagliflozin specifically increased the risk of LLA (HR: 1.19, 95% CI: 1.04–1.36, *p* = 0.01). The subgroup analysis suggested that SGLT-2i increased the risk of LLA among patients with a history of cardiovascular disease.

**Conclusion:** SGLT-2i versus oGLD was associated with a similar occurrence of LLA, UTI, and bone fracture. However, SGLT-2i was associated with a higher risk of DKA and GTI than oGLD. These findings provide valuable information on the safety profile of SGLT-2i in patients with T2DM and can help inform clinical decision-making.

## 1 Introduction

Individuals diagnosed with type 2 diabetes mellitus (T2DM) face an increased risk of mortality and cardiovascular problems. SGLT2 inhibitors (SGLT-2i) have received approval to reduce blood glucose levels in adults with T2DM when used in conjunction with diet and exercise. In addition to lowering glucose, SGLT-2i offers additional benefits in addressing various aspects of metabolic syndrome, such as improving blood pressure, weight management, and lipid profiles. Furthermore, they have shown promising outcomes in reducing cardiovascular events and overall mortality. SGLT-2i is effective in lowering cardiovascular risk in individuals with T2DM by interrupting or mitigating several processes involved in atherosclerosis development, resulting in fewer cardiovascular complications. Additionally, promising outcomes in reducing cardiovascular events and overall mortality have been reported in studies conducted by (Hu et al., 2020; Li et al., 2021; Andreadi et al., 2023). Despite their apparent advantages, a degree of controversy has arisen regarding the potential link between SGLT-2i and an elevated risk of adverse events, including diabetic ketoacidosis (DKA), lower limb amputations (LLA), genital tract infections (GTI), and others.

Several meta-analyses and cohort studies have investigated the risk of DKA, urinary tract infections (UTI), GTI, and hypoglycemia in individuals with T2DM who received SGLT-2i treatment. Multiple meta-analyses and cohort studies have been performed to evaluate the risk of DKA, UTI, or GTI in T2DM patients receiving SGLT-2i treatment. The findings have exhibited some degree of inconsistency. Concerning the risk of DKA, three meta-analyses that synthesized data from randomized controlled trials (RCTs) (Tang et al., 2016; Monami et al., 2017; Donnan et al., 2019) indicated that SGLT-2i did not increase the risk of DKA in T2DM patients. In contrast, two additional meta-analyses (Liu et al., 2020; Salah et al., 2021) reported an elevated risk of DKA in these individuals due to SGLT-2i use. Likewise, three cohort studies (Wang et al., 2017; Kim et al., 2018; Han et al., 2021) found no increased risk of DKA associated with SGLT-2i use, while three different cohort studies (Douros et al., 2020; Fralick et al., 2021a; Patorno et al., 2021) identified an increased risk of DKA in T2DM patients treated with SGLT-2i. A study by Mantovani suggested that newer hypoglycemic medications with reduced potential for drug-induced hypoglycemia can mitigate the occurrence of severe hypoglycemia and related adverse effects, particularly among more susceptible patients (Mantovani et al., 2016).

Concerning UTI and GTI, several meta-analysis of RCTs (Storgaard et al., 2016; Wu et al., 2016; Tang et al., 2017; Zhang et al., 2018) revealed that SGLT-2i increased the risk of these infections when compared to the placebo group. In contrast, two additional meta-analyses (Zhang et al., 2018; Donnan et al., 2019) reported that the incidence of UTI and GTI in the SGLT-2i group closely resembled that in the placebo group.

Multiple meta-analyses of RCTs have explored the relationship between SGLT-2i and the risk of LLA, demonstrating that SGLT-2i use elevated the LLA risk compared to the placebo group (Zaccardi et al., 2016; Rådholm et al., 2018). In contrast, additional meta-analyses (Dorsey-Treviño et al., 2020; Kumar et al., 2020) did not identify a statistically significant increase in LLA risk associated with SGLT-2i use compared to control groups. The evidence of observational studies that examine the relationship between SGLT-2i and the risk of LLA presents a broader range of findings. Seven observational studies (Chang et al., 2018; Ryan et al., 2018; Udell et al., 2018; Ueda et al., 2018; Yuan et al., 2018; Dawwas et al., 2019; Yang et al., 2019) have generated varied outcomes concerning the link between SGLT-2i use and the risk of LLA.

Observational studies using real-world data offer valuable insight into the safety and efficacy of medications in clinical practice. However, when evaluating the risk of adverse events linked to SGLT-2i, the results of these studies have displayed inconsistency. While one study (Caparrotta et al., 2021) systematically assessed the safety of SGLT-2i based on observational studies, it is important to note that the studies included were published before 2020. Because medical practices and patient demographics can evolve, the applicability of older studies in capturing current safety patterns may be constrained. Another concern is that the baseline characteristics of participants in the included studies were not balanced through propensity score matching (PSM), a statistical technique used to mitigate confounding in observational research. In the absence of PSM, there is the possibility of imbalances in the baseline characteristics between the SGLT-2i and control groups, which introduces confounding variables that might impact the study’s outcomes. Moreover, in some cases, participants in the included studies may not have been accurately classified as individuals with T2DM. Such misclassification could introduce additional confounding variables, potentially compromising the accuracy and reliability of the study’s findings. Given these constraints, conducting updated meta-analyses of real-world data and employing more robust methodologies is imperative to gain a deeper understanding of the safety profile of SGLT-2i in individuals with T2DM.

## 2 Methods

This meta-analysis followed the Preferred Reporting Items for Systematic Reviews and Meta-Analyses (PRISMA) guidelines (Kumpf et al., 2023). The protocol was registered in the International Prospective Register of Systematic Review (PROSPERO, registration number CRD42021235831).

### 2.1 Data sources

Cohort studies investigating the safety or adverse events of SGLT-2i in patients with T2DM were identified by searching the PubMed and Embase databases. All eligible studies in English published up to 11 July 2023, were considered. Literature searches used specific keywords related to SGLT-2i, such as DKA, LLA, UTI, GTI, bone fracture, hypoglycemia, safety, and adverse effects. RCTs were excluded from the search strategy. The customized search strategy for each database is shown in Supplementary Appendix S2.

### 2.2 Study selection

#### 2.2.1 Criteria for inclusion

Studies that met the following criteria were included in this meta-analysis:

1. Types of study: Prospective or retrospective cohort studies.
2. Study populations: Patients with T2DM without restrictions on age, sex, or ethnicity.
3. Study design: For retrospective cohort studies, baseline information from the observation and control groups had to be essentially the same, achieved through PSM. For prospective cohort studies, the comparability between baseline information from the observation and control groups was necessary.
4. Interventions: The observation group received treatment with SGLT-2i as a class or as individual agents, while the control group received other glucose-lowering drugs (oGLD).
5. Sample size: Studies with a sample size of 1,000 or more were included to minimize the heterogeneity of the pooled study arising from small sample size studies.

The primary outcomes were the occurrences of DKA and LLA. Secondary outcomes were the occurrences of GTI, UTI, hypoglycemia, and bone fractures. Studies were included if they reported at least one of these outcome measures.

#### 2.2.2 Criteria for exclusion

The following were the exclusion criteria for this meta-analysis:

1. RCTs, reviews, systematic reviews, meta-analyses, case reports, case studies, case series, letters, opinions, audits, protocols, and methodologies.
2. Studies in which the intervention did not meet the specified outcome measures.

Two investigators (T.T.L. and Q.Z.) independently screened the literature. In cases of discrepancies or uncertainties, consensus was reached through discussion with the other author (C.X.L.).

### 2.3 Data extraction

A standardized extraction form was used to collect the study data. The following data were independently extracted by three authors (T.T.L., Q. Z, and Q.X.): first author, year of publication, country, study population, age, sex, number of patients, intervention measure, follow-up time, and outcome measures. The data extraction forms were cross-checked to verify the accuracy and consistency of the extracted data. The third author (C.X.L.) checked all data and disagreements were resolved by discussion.

### 2.4 Quality assessment of the study

The quality assessment of the studies was conducted independently by three authors (T.T.L., Q.Z., and Q.X.) using the Newcastle-Ottawa Scale (Yu et al., 2018). This scale evaluates studies based on three main domains: selection, comparability, and exposure. Within the selection and exposure categories, each numbered item can be awarded a maximum of 1 point, while comparability can receive a maximum of 2 points. The total score ranges from 0 to 9, with higher scores indicating higher study quality. Studies were classified into low quality (scores 0–3), moderate quality (scores 4–6), and high quality (scores 7–9).

### 2.5 Statistical analysis

Meta-analysis was performed using Stata 16.0 software (StataCorp, College Station, TX, United States). Statistical heterogeneity between studies was assessed using the Cochran chi-square test complemented with the I2 statistic. *I* ^*2*^ values of 25%, 50%, and 75% indicate low, moderate, and high heterogeneity, respectively (Higgins et al., 2003). The random-effects model was used for the analysis. The hazard ratio (HR) and the 95% confidence interval (95% CI) were used to describe categorical variables, where the *p*-value < 0.05 is considered significantly different.

To examine the sources of heterogeneity, we performed a meta-regression analysis with intervention, study region, study year, sex proportion, follow-up time, and sample size as independent variables, and DKA, LLA, UTI, GTI, bone fracture, and hypoglycemia as dependent variables, respectively. To assess the stability of the results, a sensitivity analysis was systematically performed, excluding one study at a time to examine its impact on overall findings.

We utilized Confunnel plots for qualitative evaluation and Egger’s test for quantitative analysis to assess publication bias. Confunnel plots display areas of statistical significance on a funnel plot, and it help distinguish publication bias from other causes of asymmetry. If the missing studies are in areas of non-significant, the asymmetry observed in the confunnel plot is caused by publication bias. In contrast, if the absent studies are statistically significant, the observed asymmetry is more likely due to factors other than publication bias based on statistical significance (e.g., variable study quality or non-statistical significance-based publication bias mechanisms) (Peters et al., 2008). *P* < 0.05 indicates a possible publication bias.

## 3 Results

### 3.1 Literature retrieval and selection

**FIGURE 1:**
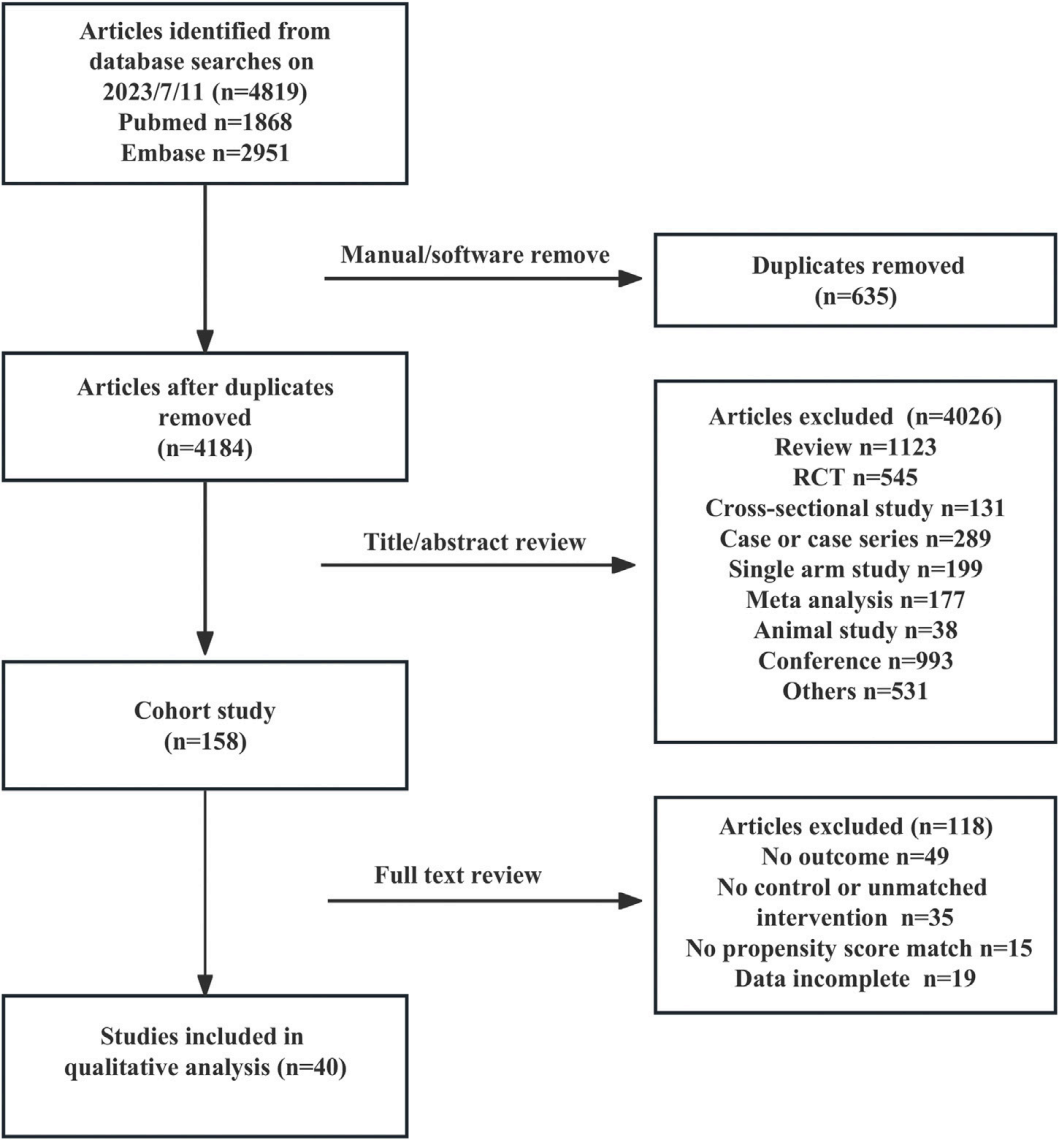
The flow chart of literature screening.

A total of 4,819 studies were retrieved, and 40 (n = 9,911,454) were included in the analysis (Birkeland et al., 2017; Nyström et al., 2017; Tang et al., 2017; Wang et al., 2017; Persson et al., 2018; Ryan et al., 2018; Toulis et al., 2018; Udell et al., 2018; Yuan et al., 2018; Kim et al., 2018; Dave et al., 2019a; Dawwas et al., 2019; Wang et al., 2019; Yang et al., 2019; Dave et al., 2019b; Norhammar et al., 2019; Douros et al., 2020; Fralick et al., 2020; Lee et al., 2020; Udell et al., 2020; Laursen et al., 2021; Yu et al., 2020; Han et al., 2021; Patorno et al., 2021; Han et al., 2021; Fralick et al., 2021b; Fralick et al., 2021b; Laursen et al., 2021; Ryan et al., 2018; Caparrotta et al., 2021; Bhosle et al., 2022; Laursen et al., 2021; Fralick et al., 2020; Schneeweiss et al., 2021, Al-et al. 2022, Ha et al., 2022; van Dalem et al., 2022; D'Andrea et al., 2023; Fu et al., 2023; Htoo et al., 2023). Figure 1 shows the literature selection flowchart.

### 3.2 Study characteristics and quality assessment

The included studies spanned from 2017 to 2023, 22 published in 2020 or later. Among these studies, 23 were conducted in the US, 7 in northern Europe, three in Canada, and 3 in Asia. The proportion of women in the included studies ranged from 33.6% to 64.7%. The median duration of follow-up was 10.8 months (3.3–58.8). Baseline glycosylated hemoglobin (HbA1c) was reported in 13 studies, while the baseline prevalence of peripheral vascular diseases (PVD) was reported in 26 studies. A comprehensive summary of the characteristics of the included studies is presented in Table 1.

**TABLE 1:** Baseline characteristics of included studies.

Study	Region	Data source	Intervention	Characteristics	Age	HbA1c† (%)	PVD ‡ (%)	Female (%)	Follow-up time	N§
Patorno et al., 2021	USA	Medicare data	SGLT-2i	age≥66	71.56±5.01		12.7	54.4	8.5m	45047
			GLP-1RA		71.56±5.03		12.9	54.0	5.5m	45047
Han et al. (2021)	Korean	KNHIS	SGLT-2i	age≥65	71.9±5.5		0.3	57.5	384.7±246.3d	15699
			DPP-4i		71.8±5.5		0.3	57.5	384.7±246.3d	15699
Fralick et al. (2021a)	Canada	ICES	SGLT-2i	age≥65	71 (68-75)	8.04 ± 1.41	2.8	40.2	1.02y	29916
			DPP-4i		71 (68-75)	8.26 ± 1.58	2.7	40.6	1.21y	29916
Yu et al., 2020	Canadian, the U.K.	CPRD	SGLT-2i	age≥18	63.8±9.5		2.2	41.6	0.90y	207817
			DPP-4i		64.0±9.6		2.2	41.9	0.88y	207817
Udell et al. (2020)	USA	MHS	Canagliflozin		65.6±8.9		15.4	43.8	2.03y	7697
			non-SGLT2i		65.7±9.7		15.5	44.7	2.03y	7697
Lee et al. (2020)	Taiwan	NHIRD	SGLT-2i	with PVD	64.7±10.7		100	50.98	0.96±0.57y	11431
			DPP-4i		65.1±14.5		100	50.49	0.66±0.45y	11431
Fralick et al. (2020)	USA	Optum	Canagliflozin	age<65 CVD (-)	51.95±8.35	8.88±1.86	0	49.4	275d	80640
			GLP-1RA		51.93±8.41	8.77±2.01	0	49.5	275d	80640
			Canagliflozin	age<65 CVD (+)	55.86±6.46	8.83±1.81	19.6	35.2		10763
			GLP-1RA		55.84±6.63	8.76±1.93	19.3	35.0		10763
			Canagliflozin	age≥65 CVD (-)	71.11±5.34	8.55±1.65	0	55.3		44522
			GLP-1RA		71.11±5.36	8.32±1.67	0	55.3		44522
			Canagliflozin	age≥65 CVD (+)	72.62±6.17	8.49±1.63	21.3	42.5		19495
			GLP-1RA		72.60±6.08	8.40±1.65	21.1	42.7		19495
Douros et al. (2020)	Canadian, the U.K.	CPRD	SGLT-2i		63.8±9.5		2.3	41.4	0.9±0.8y	208757
			DPP-4i		64.0±9.5		2.3	41.6	0.9±0.8y	208757
Yang et al. (2019)	USA	CCAE	SGLT-2i		52.5 ± 8.2		2.8	47.2	0.42 (0.21–0.76)y	49324
			DPP-4i		52.3±8.2		2.9	49.2	0.43 (0.22–0.91)y	50189
			SGLT-2i		52.3±8.2		2.8	47.8	0.42 (0.21–0.76)y	46878
			SU		52.3±8.1		2.9	48.9	0.43 (0.22–0.91)y	48954
Wang et al. (2019)	USA	CCAE/MDCD/MDCR/Optum	SGLT-2i		47.9-75.5			41.0-72.1		131542
			SU							131542
			DPP-4i							149775
			GLP-1RA							123792
			TZD							62084
			insulin							92890
			metformin							73994
Fralick et al. (2019)	USA	Optum	Canagliflozin		56.3110.89	8.76±1.79	4.5	45.5	247±191d	23458
		Optum	GLP-1RA		56.04±11.47	8.72±1.92	4.5	45.4	224±185d	23458
		MarketScan	Canagliflozin		54.53±9.79	8.74±1.82	3.2	49.3		56506
			GLP-1RA		54.55±10.00	8.60±1.90	3.2	49.4		56506
Dawwas et al. (2019)	USA	MarketScan	SGLT-2i		54±12.4			47.7	1.00y	62767
			SU		54±9.6			47.6	1.00y	62767
			SGLT-2i		55±9.2			46.1		66633
			DPP-4i		54±11.0			46.2		66633
Dave et al., 2019a	USA	MarketScan/Optum	SGLT-2i			8.8±1.9		45.4	F (150/145)d M(168/154d	143034
			DPP-4i/GLP-1RA			8.8±1.8		45.4	F (150/145)d M(168/154d	143034
Dave et al., 2019a	USA	MarketScan	SGLT-2i		54.7±9.90	8.8±1.8	1.7	45.9	204d	61876
			DPP-4i		54.7±10.2	8.8±1.9	1.9	45.6	190d	61876
		Optum	SGLT-2i		54.5±10.1	8.7±1.8	1.6	50.6	221	55989
			GLP-1RA		54.4±10.1	8.8±1.9	1.6	50.5	191d	55989
Adimadhyam et al. (2019)	USA	MarketScan	SGLT-2i		54.78±9.90			47.42	219d	30549
			DPP-4i		54.66±10.55			47.23	47.23	30549
Yuan et al. (2018)	USA	CCAE	Canagliflozin		53.2±8.1		8.1	44.5	1.27	63845
			non-SGLT2i		53.3±8.1		8.2	44.7	1.04y	63845
Udell et al. (2018)	USA	MHS	SGLT-2i		65.8±8.9		16.3	43.3	1.7y	12629
			non-SGLT2i		65.9±9.8		15.4	44.9	1.5y	12629
Toulis KA (2018)	UK	THIN	Dapagliflozin	age≥40	59.4±9.4			56.7	12.9±8.4m	4548
			non-SGLT2i		59.4±9.4			56.7	11.9±8.2m	18070
Persson et al. (2018)	Denmark, Norway		Dapagliflozin		61±11.1			41.0	0.95y	10227
	Sweden		DPP-4i		60.8±12.4			40.4	0.95y	0.95y
Kim et al., (2018)	Korean		SGLT-2i		53.1211.81		0.59	44.9	319.1d	56325
			DPP-4i		53.33±12.32		0.55	44.7	319.1d	56325
Chang et al. (2018)	USA	MarketScan	SGLT-2i		53.5±8.5			47.3	115	39869
			DPP-4i		54.5±8.4			43.3	121d	105023
			GLP-1RA		51.9±9.5			61.0	99d	39120
			oGLD		51.4±11.3			55.6	127d	769894
Wang et al. (2017)	USA	CCAE	SGLT-2i		55.0±6.6					27515
			oGLD		55.0±6.5					27515
Birkeland et al. (2017)	Denmar, Norway, Sweden		SGLT-2i		61.2±10.9			40.6	0.9 ± 4.1y	22830
			oGLD		61.2±12.4			39.5	0.9 ± 4.1y	68490
Norhammar et al. (2019)	Sweden		Dapagliflozin	age≥40	66.3±7.5		6.2	33.6	1.6y	7102
			oGLD		66.2±8.1		6.3	33.8	1.6y	21306
Nyström et al. (2017)	Sweden	ATC	Dapagliflozin		61.2±10.4		3	38	1.51y	6139
			insulin		61.1±12.8		3	37	1.4y	6139
Pasternak et al. (2019)	Denmar, Norway, Sweden		SGLT-2i	age 35–84	61±10			40	1.1y	20983
			DPP-4i		61±10			40	1.7y	
Ryan et al. (2018)	USA	CCAE	Canagliflozin		45.4		7.9	45.4		60073
			oGLD		45.3		8.1	45.3		217583
		MDCD	Canagliflozin		65.0		13.5	64.7		5,998
			oGLD		64.4		13.7	65.0		38172
		MDCR	Canagliflozin		42.7		23.8	42.7		9206
			oGLD		42.9		24.1	42.9		42744
		Optum	Canagliflozin		44.1		13.8	44.1		42744
			oGLD		43.7		13.8	43.7		146868
		CCAE	Empagliflozin or Dapagliflozin							54324
			oGLD							175500
		MDCD	Empagliflozin or Dapagliflozin							1,378
			oGLD							25995
		MDCR	Empagliflozin or Dapagliflozin							4336
			oGLD							31228
		Optum	Empagliflozin or Dapagliflozin							19588
			oGLD							118027
Dawwas et al. (2022)	USA	Optum	SGLT-2i	with PVD	60.711.0		7.0	57.2	131d	85125
			DPP-4i		60.4±12.1		7.0	57.4	131d	
Fralick et al. (2021b)	USA	MarketScan	SGLT-2i		53.50±10.00	1.81		51.5	147d	9964
			metformin		53.51±11.50	1.64		51.5	213d	9964
Ha et al. (2022)	Korean	KNHIS	SGLT2i	with PVD	54.80±12.30		19.8	41.2		42230
			DPP-4i		54.80±12.30		19.7	41.2		
Laursen et al. (2021)	Denmar, Norway, Sweden		SGLT-2i		61.3±11.2			37.9		10923
			GLP-1RA		57.5±12.0			44.2		18849
Paul et al. (2021)	USA	CEMR	SGLT-2i	with PVD	57.5±10.8	8.6±1.7	6	47	1.8	169739
			GLP-1RA		56.8±11.8	8.2±1.9	4	60	3.4y	149826
			DPP-4i		62.8±11.4	8.2±1.8	6.0	50.0	3.3y	448225
			oGLD		60.6±12.1	7.8±2.0	4.0	51.0	4.9y	1954353
Werkman et al. (2021)	Netherlands	GOLD	SGLT-2i	with PVD	57.8±10.5	8.8±1.6	1.4	42.6	3.0y	10927
			SU		61.6±13.2	8.7±1.8	2.0	42.8	4.4y	
Zerovnik et al. (2022)	Slovenia		SGLT-2i	age≥40	64.04±8.8			40.8		2939
			DPP-4i		63.99±8.9			39.5		
Patorno et al. (2022)	USA	Optum MarketScan	Empagliflozin	with PVD	60.23	8.49±1.78	5.9	45.4	6m	39072
			DPP-4i		60.32	8.55±1.84	5.8	45.5	6m	39072
Al-Mashhadi ZK et al. (2022)	Denmark, Norway, Sweden		SGLT-2i		61.1±11.3			38.5	335	9190
			GLP-1RA		58.5±12.0			40.0	372d	9190
Dalem et al., 2022	UK	CPRD	SGLT-2i		58.2±10.2	8.8±1.6		43	2.4y	6579
			SU		62.0±12.9	8.6±1.7		42.7	3.4y	13767
D'Andrea et al. (2023)	USA	Optum	SGLT-2i		60.6±11.7	8.7±1.0		45.7	8m	43637
			DPP-4i		60.6±11.6	8.7±1.0		45.7	8m	43637
Fu et al. (2023)	USA	Optum	SGLT-2i	with PVD	72.29±7.39		18.6	44.3	7.5m	28847
			GLP-1RA		72.32±7.40		18.8	44.3	7.5m	28847
Htoo et al. (2023)	USA	MarketScan	SGLT-2i	with PVD	71.8±5.1		12.9	49.8	7m	82994
			DPP-4i		71.8±5.1		12.9	50	7m	82994
			SGLT-2i	with PVD	71.8±5.2		13.9	53.2	7m	88726
			GLP-1RA		71.9±5.2		14.0	53.2	7m	88726

Legend: † HbA1c = Glycated hemoglobin A1c, ‡ PVD, peripheral vascular diseases, §N = sample size, m = month, d = day, y = year, SGLT-2i = sodium-glucose cotransporter 2 inhibitors, oGLD, other glucose lowering drugs, GLP-1RA = GLP-1, receptor agonist, DPP-4i = dipeptidyl peptidase 4 inhibitors, TZD, thiazolidinedione; SU, sulfonylureas; CVD, cardiovascular disease.

### 3.3 Primary outcomes

#### 3.3.1 DKA

Fourteen studies compared the safety of the SGLT-2i class and oGLD in terms of DKA among the 40 trials that met the inclusion criteria (Kim et al., 2018; Han et al., 2021; Fralick et al., 2021a; Douros et al., 2020; Patorno et al., 2021; Tang et al., 2017; Laursen et al., 2021; Fralick et al., 2021b; Toulis et al., 2018; Patorno et al., 2021; Laursen et al., 2021; Bhosle et al., 2022; D'Andrea et al., 2023; Fu et al., 2023). A total of 2,665 DKA cases were reported among 1,233,569 patients treated with SGLT-2i (mean incidence rate 2.86 per 1,000 person-years). In contrast, 2,063 DKA cases were reported among 1,306,053 patients treated with oGLD (mean incidence rate DKA 1.87 per 1,000 person-years) (Supplementary Appendix S4.1).

**FIGURE 2:**
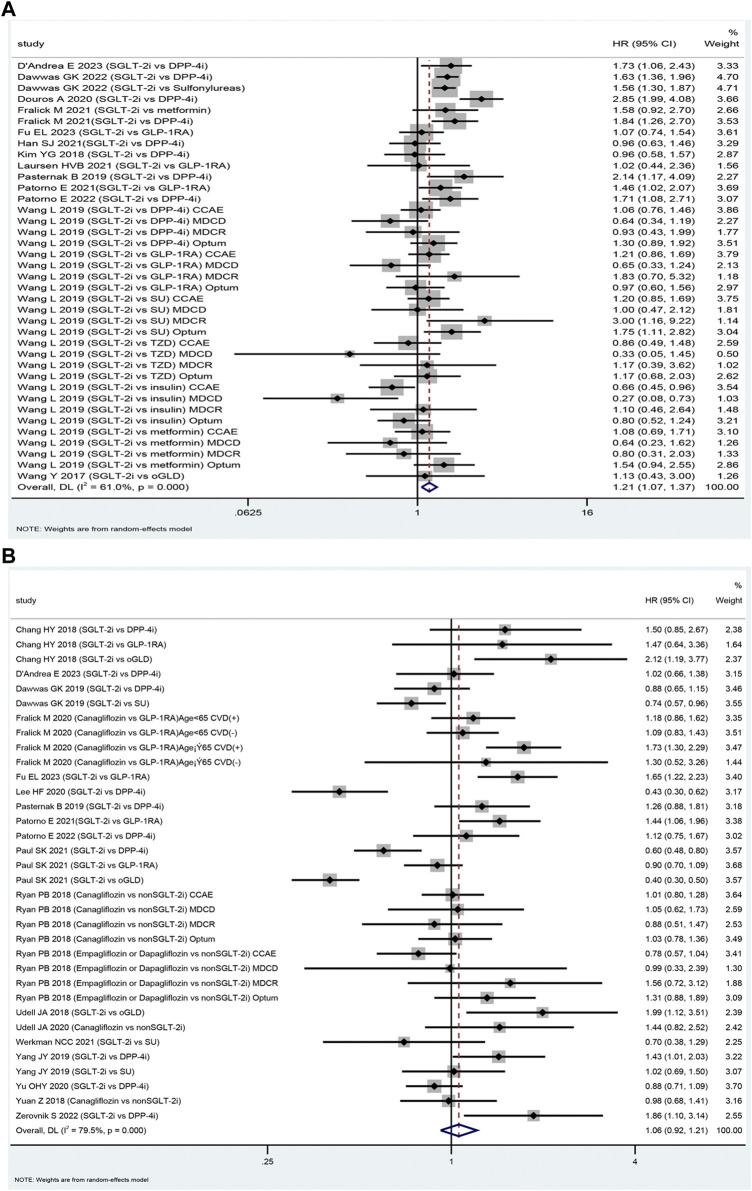
The forest plot of diabetic ketoacidosis **(A)** and lower limb amputation **(B)** Legend: SGLT-2i = sodium-glucose cotransporter 2 inhibitors, GLP-1RA = GLP-1 receptor agonist, DPP-4i = dipeptidyl peptidase 4 inhibitors, SU = sulfonylureas, TZD = thiazolidinedione, oGLD = other glucose-lowering drugs, CVD = cardiovascular disease.

The SGLT-2i class was associated with an increased risk of DKA compared to oGLD (HR: 1.21, 95% CI: 1.07–1.38, *p* = 0.003, I^2^ = 61.0%; Figure 2A). Subgroup analysis revealed that the SGLT-2i class did not significantly increase the risk of DKA compared to glucagon-like peptide-1 receptor agonists (GLP-1RA). Among 208,609 patients, there were 393 DKA events in the SGLT-2i group and 348 in the GLP-1RA group [mean incidence rates 2.20 *vs*. 1.76 per 1,000 person-years (Supplementary Appendix S4.1); HR: 1.15; 95% CI: 0.96–1.37; *p* = 0.125; I^2^ = 3.4% (Supplementary Appendix S5.1). However, the analysis found an increased risk of DKA in the SGLT-2i group compared to DPP-4i. Among 627,019 patients, there were 1,224 DKA events in the SGLT-2i group and 799 events in the DPP-4i group [mean incidence rates 2.46 *vs*. 1.48 per 1,000 person-years (Supplementary Appendix S4.1); HR: 1.38; 95% CI: 1.10–1.74; *p* = 0.006; *I* ^*2*^ = 72.8% (Supplementary Appendix S5.1).

Among 224,186 patients, canagliflozin had 653 DKA events compared to 376 events with DPP-4i [mean incidence rate 4.93 *vs*. 2.54 per 1,000 person-years; HR: 1.57; 95% CI: 1.12–2.19; *p* = 0.008; *I* ^*2*^ = 67.4%]. Among 96, 077 patients, dapagliflozin had 171 DKA events compared to 117 events with DPP-4i [mean incidence rate 5.17 vs*.* 1.94 per 1,000 person-years; HR: 1.54; 95% CI: 1.14–2.087; *p* = 0.004; *I* ^*2*^ = 0.0%]. Among 151,625 patients, empagliflozin had 241 DKA events compared to 192 events with DPP-4i [mean incidence rate 3.16 *vs*. 2.03 per 1,000 person-years; HR: 1.50; 95% CI: 1.14–1.97; *p* = 0.004; *I* ^*2*^ = 23.7%]. Details are shown in Supplementary Appendix S5.2.

#### 3.3.2 LLA

Eighteen studies were included in the assessment of the safety of SGLT-2i versus oGLD in terms of LLA (Fralick et al., 2021b; Fralick et al., 2019; Yang et al., 2019;Ueda et al., 2018; Udell et al., 2018; Ryan et al., 2018; Dawwas et al., 2019; Chang et al., 2018; Caparrotta et al., 2021; Wang et al., 2019; Persson et al., 2018; Udell et al., 2020; Wang et al., 2019; Dawwas et al., 2019; Dave et al., 2019a; Dave et al., 2019b; D'Andrea et al., 2023; Fu et al., 2023). Among these studies, 2,025 LLA cases were reported in the SGLT-2i group (mean incidence rate 2.30 per 1,000 person-years). In comparison, the oGLD group had 4,142 LLA cases (mean incidence rate of 1.98 per 1,000 person-years) (Supplementary Appendix S4.2). The pooled analysis indicated that the use of SGLT-2i was not associated with a significantly higher risk of LLA compared to oGLD (HR: 1.06, 95% CI: 0.92–1.21, *p* = 0.42, *I* ^*2*^ = 79.5%; Figure 2B).

The meta-analysis showed that the SGLT-2i class increased the risk of LLA compared to GLP-1RA (629 *vs*. 442 events among 269,183 patients; mean incidence rate 5.28 *vs*. 3.56 per 1,000 person-years; HR: 1.22; 95% CI: 1.00–1.45; *p* = 0.048; *I* ^*2*^ = 72.5%; Supplementary Appendix S5.3). However, there was no significant difference in LLA risk between the SGLT-2i class and DPP-4i (670 *vs*. 830 events among 471,139 patients; mean incidence rate 3.33 *vs*. 2.75 per 1,000 person-years; HR: 0.98, 95% CI: 0.77–1.26; *p* = 0.89; *I* ^*2*^ = 81.2%; Supplementary Appendix S5.3). In contrast, the SGLT-2i class showed a lower risk of LLA compared to sulfonylurea (164 events among 122,648 patients *vs*. 415 events among 131,372 patients; mean incidence rate 1.46 *vs*. 1.70 per 1,000 person-years; HR: 0.80, 95% CI: 0.65–0.99; *p* = 0.04; *I* ^*2*^ = 1.40%; Supplementary Appendix S5.3).

Canagliflozin increased the risk of LLA compared to oGLD (HR: 1.19; 95% CI: 1.04–1.36, *p* = 0.01, *I* ^*2*^ = 36.8%; Supplementary Appendix S5.4). Regarding the prevalence of PVD in the included studies, the analysis revealed that SGLT-2i did not increase the risk of LLA when the baseline PVD prevalence was less than 10% (1,190 events among 735,546 patients *vs*. 3,093 events among 1,324,812 patients; mean incidence rate 1.66 *vs*. 1.61 per 1,000 person-years; HR: 0.87; 95% CI: 0.72–1.06; *p* = 0.18; *I* ^*2*^ = 79.8%; Supplementary Appendix S5.5). The prevalence of PVD in these studies ranged from 0% to 8.1% (Ryan et al., 2018; Yang et al., 2019; Yuan et al., 2018; Fralick et al., 2020; Yu et al., 2020; Paul et al., 2021; Werkman et al., 2021; Patorno et al., 2022). Similarly, when the baseline PVD prevalence was greater than 10% (768 events among 264,885 patients *vs*. 2,347 events among 845,427 patients; mean incidence rate 7.39 *vs*. 5.42 per 1,000 person-years), SGLT-2i was not associated with an increased risk of LLA (HR: 1.21, 95% CI: 0.96–1.52, *p* = 0.22, *I* ^*2*^ = 75.8%; Supplementary Appendix S5.5). The prevalence of PVD in these six studies ranged from 12.7% to 23.8% (Ryan et al., 2018; Udell et al., 2018; Fralick et al., 2020; Lee et al., 2020; Udell et al., 2020; Patorno et al., 2021; Fu et al., 2023).

In patients with previous cardiovascular disease, SGLT-2i increased the risk of LLA compared to oGLD (HR: 1.24, 95% CI: 1.05–1.46, *p* = 0.046, *I* ^*2*^ = 37.6%; Supplementary Appendix S5.6). Specifically, there were 772 LLA events among 248,685 patients in the SGLT-2i group (mean incidence rate 8.42 per 1,000 person-years). In comparison, the oGLD group had 2,350 LLA events among 829,227 patients (mean incidence rate 5.91 per 1,000 person-years). In contrast, SGLT-2i did not increase the risk of LLA in patients without cardiovascular disease at baseline compared to oGLD (HR: 0.90, 95% CI: 0.48–1.68, *p* = 0.74, *I* ^*2*^ = 83.2%, Supplementary Appendix S5.6). There were 198 LLA events among 125,162 patients in the SGLT-2i group (mean incidence rate 2.01 per 1,000 person-years). In comparison, the oGLD group had 151 LLA events among 125,162 patients (mean incidence rate 1.58 per 1,000 person-years).

### 3.4 Secondary outcomes

In the SGLT-2i group, 615 cases of UTI (mean incidence rate 9.58 per 1,000 person-years), 10,178 GTI cases (median incidence rate 58.67 per 1,000 person-years), 3,751 cases of bone fractures (mean incidence rate 5.18 per 1,000 person-years), and 302 cases of hypoglycemia (mean incidence rate 6.58 per 1,000 person-years) were identified (Supplementary Appendix S4.3-4.5). In comparison, the oGLD group had 305 cases of UTI (mean incidence rate 7.31 per 1,000 person-years), 2,390 cases of GTI (median incidence rate 15.64 per 1,000 person-years), 1,299 cases of bone fractures (mean incidence rate 4.34 per 1,000 person-years), and 2,167 cases of hypoglycemia (mean incidence rate 9.42 per 1,000 person-years) (Supplementary Appendix S4.3-4.5).

**FIGURE 3:**
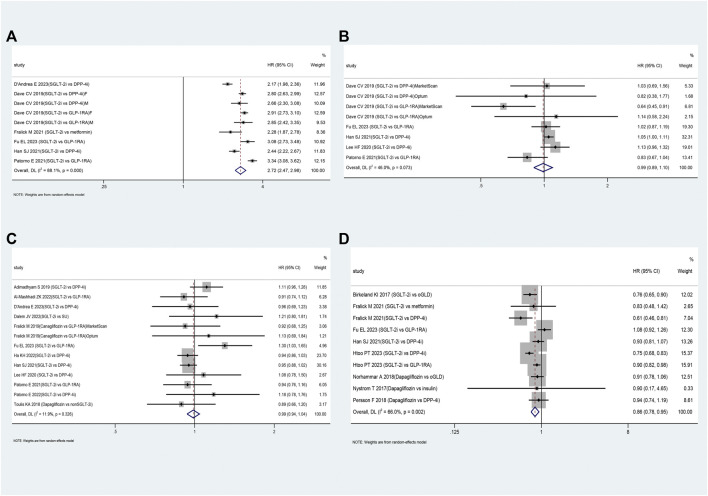
The forest plot of secondary outcomes. Legend: **(A)**: genital tract infections; **(B)**: urinary tract infections; **(C)**: bone fractures; **(D)**: hypoglycemia. SGLT-2i = sodium-glucose cotransporter 2 inhibitors, GLP-1RA = GLP-1 receptor agonist, DPP-4i = dipeptidyl peptidase 4 inhibitors, SU = sulfonylureas, oGLD = other glucose-lowering drugs, F=female, M=male.

Table 2 presents the pooled results of various outcomes from multiple studies. The use of SGLT-2i was not associated with an increased risk of UTI (HR: 0.99, 95% CI: 0.89–1.10, *p* = 0.83; Figure 3B) based on these studies (Dave et al., 2019b; Lee et al., 2020; Han et al., 2021; Patorno et al., 2021; D'Andrea et al., 2023; Fu et al., 2023) or bone fractures (HR: 0.99, 95% CI: 0.94–1.04, *p* = 0.66; Figure 3C) compared to oGLD based on these studies (Toulis et al., 2018; Adimadhyam et al., 2019; Fralick et al., 2019; Lee et al., 2020; Han et al., 2021; Patorno et al., 2021; Al-Mashhadi et al., 2022; Ha et al., 2022; Patorno et al., 2022; van Dalem et al., 2022; D'Andrea et al., 2023; Fu et al., 2023). However, SGLT-2i showed a 2.72-fold increase in GTI risk (HR: 2.72, 95% CI: 2.47–2.98, *p* < 0.01; Figure 3A) based on these studies (Dave et al., 2019b; Fralick et al., 2021a; Han et al., 2021; Patorno et al., 2021; D'Andrea et al., 2023; Fu et al., 2023) and a reduced risk of hypoglycemia (HR: 0.86, 95% CI: 0.78–0.95, *p* = 0.002; Figure 3D) compared to oGLD based on these studies (Birkeland et al., 2017; Nyström et al., 2017; Persson et al., 2018; Norhammar et al., 2019; Fralick et al., 2021b; Han et al., 2021; Htoo et al., 2023).

**TABLE 2:** Results of secondary outcomes.

Outcome^†^	Group^‡^	Studies included	I^2^	HR	95% CI	P
UTIs	SGLT-2i vs oGLD	4	46.0	0.99	0.89,1.10	0.83
	SGLT-2i vs GLP-1RA	2	25.9	0.79	0.63,1.00	0.049^*^
	SGLT-2i vs DPP-4i	3	0.0	1.06	1.01,1.11	0.03^*^
GTIs	SGLT-2i vs oGLD	6	88.1	2.72	2.47,2.98	<0.01^**^
	SGLT-2i vs oGLD(F)	4	82.8	3.11	2.81,3.44	<0.01^**^
	SGLT-2i vs oGLD(M)	4	80.9	3.02	2.48,3.68	<0.01^**^
	SGLT-2i vs GLP-1RA	4	60.8	2.76	2.50,3.04	<0.01^**^
	SGLT-2i vs DPP-4i	4	86.6	2.50	2.21,2.84	<0.01^**^
Bone fracture	SGLT-2i vs oGLD	11	11.9	0.99	0.94,1.04	0.66
	SGLT-2i vs DPP-4i	4	23.2	1.00	0.92,1.09	0.99
	SGLT-2i vs GLP-1RA	3	40.2	1.06	0.88,1.27	0.56
hypoglycemia	SGLT-2i vs oGLD	7	66.0	0.86	0.78,0.95	0.02^**^

Legend: † UTIs, urinary tract infections; GTIs, genital tract infections, ‡ SGLT-2i = sodium-glucose cotransporter 2 inhibitors, oGLD, other glucose lowering drugs, GLP-1RA = GLP-1, receptor agonist, DPP-4i = dipeptidyl peptidase 4 inhibitors, F = female, M = male.

Subgroup analysis revealed that SGLT-2i increased the risk of UTI by approximately 6% compared to DPP-4i (HR: 1.06, 95% CI: 1.01–1.11, *p* < 0.01; Supplementary Appendix S5.7). Compared to DPP-4i and GLP-1RA, SGLT-2i showed a 2.50-fold and 3.06-fold increased risk of GTI, respectively (Supplementary Appendix S5.8). Furthermore, the risk of GTI associated with SGLT-2i was consistent between patients of different sex (Supplementary Appendix S5.9). However, SGLT-2i was not associated with an increased risk of bone fractures compared to DPP-4i or GLP-1RA (Supplementary Appendix S5.10).

### 3.5 Meta-regression

Meta-regression indicated that none of the above independent variables was identified as the source of heterogeneity (Supplementary Appendix S6). For the subgroup analysis of DKA and LLA, we conducted separate evaluations based on different study regions (Supplementary Appendix S9). In studies in northern Europe, SGLT-2i did not show a significant increase in the risk of DKA (HR: 1.56, 95% CI: 0.76–3.20, *p* = 0.22). However, studies in the US showed a slightly elevated risk of DKA associated with SGLT-2i use (HR: 1.16, 95% CI: 1.02–1.31, *p* = 0.022). Similarly, studies conducted in Canada revealed a significantly higher risk of DKA with SGLT-2i use (HR: 2.30, 95% CI: 1.50–3.53, *p* < 0.01). Regarding LLA risk, SGLT-2i did not demonstrate a significant increase in the US (HR: 1.12, 95% CI: 0.92–1.35, *p* = 0.26) or in northern Europe (HR: 1.03, 95% CI: 0.91–1.16, *p* = 0.63).

### 3.6 Sensitivity analysis

Sensitivity analysis demonstrated that the combined effect values remained consistent before and after excluding any study for the above outcomes (Supplementary Appendix S7). This consistency suggests that the study results were stable.

### 3.7 Publication bias analysis

Confunnel plots for DKA, genital infections, and hypoglycemia showed that missing studies were in areas of statistical significance, which suggests that asymmetry is more likely to be due to factors other than publication bias. Confunnel plots for LLA revealed that absent studies were distributed across regions of statistical significance, as well as those yielding inconclusive results, suggesting that the detected asymmetry may be attributable to publication bias and other confounding factors. (Supplementary Appendix S8). Regarding Egger’s test, the *p* values for UTI, bone fracture, GTI, and hypoglycemia were 0.27, 0.14, 0.39, and 0.93, respectively, suggesting no significant publication bias, except for LLA (*p* = 0.045) and DKA (*p* = 0.01).

## 4 Discussion

A total of 9,911,454 patients with T2DM were included in this real-world data study. When assessing SGLT-2i as a class and as individual agents, DPP-4i and GLP-1RA were the two most used active comparators.

The mean or median incidence rates per 1,000 person-years for various adverse events in the SGLT-2i group were the following: 2.86 for DKA, 2.30 for LLA, 9.58 for UTI, 15.64 for GTI, 5.18 for bone fractures, and 9.42 for hypoglycemia. Compared to the control group, the mean incidence rates of DKA and GTI were significantly higher in the SGLT-2i group. In contrast, the rates of LLA, UTI, bone fractures, and hypoglycemia were similar to those of the control group. In a meta-analysis of five RCTs, the mean spontaneous rates of DKA, GTI, and amputations were 0.3, 4, and 4 per 1,000 person-years, respectively (Marilly et al., 2022). Furthermore, a matched incidence rate ranging from 33.58 to 35.66 per 1,000 person-years for GTI was reported in five comparative cohorts (Alkabbani et al., 2022). The absolute rate of DKA ranged from 0.6 to 2.2 per 1,000 person-years in RCTs and ranged from 0.6 to 4.9 per 1,000 person-years in observational studies (Colacci et al., 2022). Interestingly, our real-world study indicated that adverse events such as DKA and GTI were slightly higher than observed in RCTs.

The SGLT-2i class was associated with an increased risk of DKA by approximately 21% compared to oGLD (Colacci et al., 2022). However, compared to GLP-1RA, the SGLT-2i class did not show an increased risk of DKA. On the other hand, compared to DPP-4i, the SGLT-2i class increased the risk of DKA. The subgroup analysis also revealed that specific SGLT-2 inhibitors, namely canagliflozin, empagliflozin, and dapagliflozin, could increase the risk of DKA compared to DPP-4i. Interestingly, SGLT-2i increased the risk of DKA 2.3 times in Canadians but not in patients from Nordic countries. The reason for this disparity can be attributed to the fact that the two studies conducted in Canada (Douros et al., 2020; Fralick et al., 2021b) compared SGLT-2i with DPP-4i, while the two studies in Nordic countries compared SGLT-2i with both DPP-4i and GLP-1RA. Our findings are consistent with previous studies (Colacci et al., 2022; Marilly et al., 2022).

Studies have indicated a higher risk of DKA in older patients and those with a history of insulin use or poor glycemic control (Fralick et al., 2021a). However, a subgroup analysis of these factors was not feasible due to the limited data provided by the included studies on baseline insulin use, fasting glucose levels, postprandial glucose levels, and HbA1c values.

The CANVAS trial reported that the use of canagliflozin increased the risk of LLA nearly twice (HR 1.97; 95% CI 1.41–2.75) compared to placebo, raising concerns about the possible risk of LLA with SGLT-2i (Neal et al., 2017). We did not find an increased risk of LLA with the SGLT-2i class compared to oGLD, DPP-4i, or sulfonylureas. However, we observed that patients taking canagliflozin had a 19% higher LLA risk than those taking oGLD. Unfortunately, due to limited data, we could not analyze the association between dapagliflozin or empagliflozin and the risk of LLA.

A previous study revealed that preexisting PVD was the main factor influencing the risk of amputation (Paul et al., 2021). Although the studies included in our analysis did not support grouping based on the presence or absence of previous PVD, we performed a subgroup analysis categorizing baseline PVD prevalence into two groups: PVD incidence <10% and PVD incidence >10%. The results of the subgroup analysis indicated that SGLT-2i decreased the risk of LLA by 13% among patients with a preexisting PVD incidence <10%, while it increased the risk of LLA by 21% among patients with a PVD incidence >10%. However, although these differences were not statistically significant, the findings were consistent with the analysis results conducted on the entire study group. Furthermore, we also examined patients with and without cardiovascular disease at baseline as subgroups. The analysis suggested that SGLT-2i increased the risk of LLA in patients with cardiovascular disease at baseline. However, these subgroup analysis results should be interpreted cautiously as they were based on fewer studies (3–4) included. More research and verification are required to confirm these findings.

Studies have indicated that preexisting LLA can increase the risk of future LLA (Arnott et al., 2020). In the studies we included, researchers matched the baseline incidence of LLA between the groups using PSM or directly excluded patients with preexisting LLA. This approach helps minimize the influence of this confounding factor on our results. However, it is crucial to acknowledge that publication bias could potentially affect estimates of the association between the use of SGLT2i and the risk of LLA.

This study showed that SGLT-2i did not increase the risk of UTI or bone fractures. Several meta-analyses of RCTs have consistently shown that the risk of UTI did not increase with SGLT-2i compared to the placebo or active comparator groups (Storgaard et al., 2016; Wu et al., 2016; Puckrin et al., 2018). However, a subgroup analysis revealed a 6% increased risk of UTI when SGLT-2i was compared to DPP-4i. It is essential to interpret this result with caution, as it was mainly influenced by a study of a Korean study conducted in 2021 (Han et al., 2021). This study included older patients over 65 years of age, with a significant proportion of women (57.5%). T2DM itself is a known risk factor for UTI, regardless of the treatment regimen (Nitzan et al., 2015), and older women are particularly susceptible to UTI (Nicolle et al., 2014; Fioretto et al., 2016). Additionally, the study did not match the baseline incidence of UTI. Therefore, we must consider these specific factors and potential confounders when interpreting the association between SGLT-2i use and the risk of UTI in our study.

Our study revealed that SGLT-2i did not increase the risk of bone fractures, even in patients with T2DM over 65 years of age. These findings are consistent with the results of a previous study (Wu et al., 2016). However, concerning GTI, SGLT-2i use was associated with a three-fold increased risk, regardless of sex. This result remained consistent across different subgroup analyses based on various active comparators. Several meta-analyses have also reported an increased risk of GTI with SGLT-2i (Jabbour et al., 2018; Zhang et al., 2018). Fortunately, GTI is considered an adverse event of lesser severity than other safety outcomes and can be mitigated by educating patients to increase their water intake during medication. Furthermore, the hypoglycemic effect of SGLT-2i is independent of insulin, resulting in a 14% lower risk of hypoglycemia than other medications.

## 5 Strength and limitation of the study

Our investigation has a large cohort of 9,911,454 patients with T2DM and included data from 40 cohort studies conducted in various countries. Rigorous inclusion and exclusion criteria were used to ensure the robustness of the study. The PSM matched the baseline information of included studies and some excluded patients with preexisting LLA, further reducing potential confounding variables and increasing the reliability of our results. To address the study heterogeneity, we used meta-regression and subgroup analysis. Fortunately, our sensitivity analyses did not show significant differences compared to our primary analyses, reinforcing the reliability of our findings. We also assessed potential publication bias using Egger’s test, which indicated no evidence of bias except for LLA and DKA.

However, our study has the following limitations. First, although all included studies had comparable demographic characteristics between treatment groups through PSM, there may still be residual confounding from some unmeasured or not fully measured factors (e.g., HbA1c level, diabetes duration, prior insulin use) that cannot be completely ruled out. Second, certain pooled studies showed high heterogeneity. Third, the discussion of the safety of SGLT-2i as an individual agent was limited due to the availability of limited data. Furthermore, the subgroup analysis included only a few studies included, and inconsistencies in the results of some subgroup analyses must be verified by more high-quality studies with larger sample sizes.

## 6 Conclusion

In a comprehensive population-based cohort study comprising 9,911,454 patients with T2DM, the use of SGLT-2i compared to oGLD showed a similar incidence of LLA, UTI, and bone fractures. However, SGLT-2i was associated with a higher risk of DKA and GTI than oGLD. The subgroup analysis indicated that the use of SGLT-2i was associated with an increased risk of LLA among patients with a history of CVD. Specifically, canagliflozin, empagliflozin, and dapagliflozin increased the risk of DKA compared to DPP-4i. Canagliflozin was associated with an elevated risk of LLA.

## Data Availability

The datasets presented in this study can be found in online repositories. The names of the repository/repositories and accession number(s) can be found in the article/Supplementary Material.
