# Supplementary material for: Safety of sodium-glucose transporter 2 (SGLT-2) inhibitors in patients with type 2 diabetes: a meta-analysis of cohort studies
Source: Front Pharmacol. 2023 Oct 13;14:1275060. doi: 10.3389/fphar.2023.1275060 (PMC10613530; doi:10.3389/fphar.2023.1275060)
Supplement: Supplementary file 1 [file DataSheet1.docx]

**Content**

**[Appendix 1: PRISMA checklist](#_Toc26430)**

**[Appendix 2. Search strategy](#_Toc8678)**

**[Take Pubmed for example](#_Toc20182)**

**[Appendix 3. Risk of Bias Assessment](#_Toc10753)**

**[Appendix 4: The events and incidence rate of outcomes](#_Toc18553)**

**[4.1 The events and incidence rate of diabetic ketoacidosis](#_Toc6996)**

**[4.2 The events and incidence rate of lower limb amputation](#_Toc7533)**

**[4.3 The events and incidence rate of urinary tract infections](#_Toc22233)**

**[4.4 The events and incidence rate of genital tract infections](#_Toc7263)**

**[4.5 The events and incidence rate of hypoglycemia](#_Toc9121)**

**[Appendix 5: Forest plot for meta-analysis](#_Toc16080)**

**[5.1 Forest plot of SGLT-2i vs DPP-4i/GLP-1RA for diabetic ketoacidosis](#_Toc28943)**

**[5.2 Forest plot of Canagliflozin/Dapagliflozin/Empagliflozin vs DPP-4i for diabetic ketoacidosis](#_Toc19160)**

**[5.3 Forest plot of SGLT-2i vs DPP-4i/GLP-1RA/SU for lower limb amputations](#_Toc14366)**

**[5.4 Forest plot of Canagliflozin compared with other glucose-lowering drugs for lower limb amputations](#_Toc23515)**

**[5.5 Forest plot for lower limb amputations according to the prevalence of peripheral vascular diseases](#_Toc23462)**

**[5.6 Forest plot for lower limb amputations in patients with or without prior cardiovascular disease](#_Toc21041)**

**[5.7 Forest plot of SGLT-2i vs DPP-4i/GLP-1RA for urinary tract infections](#_Toc10235)**

**[5.8 Forest plot of SGLT-2i vs DPP-4i/GLP-1RA for genital tract infections](#_Toc26833)**

**[5.9 Forest plot of genital tract infections in female and male patients](#_Toc21598)**

**[5.10 Forest plot of SGLT-2i vs DPP-4i/GLP-1RA for bone fracture](#_Toc27001)**

**[Appendix 6: Results of meta regression analysis (](#_Toc15637)*[P](#_Toc15637)*^[†](#_Toc15637)^[)](#_Toc15637)**

**[Appendix 7: Results of sensitive analysis](#_Toc31465)**

**[7.1 Sensitive analysis of diabetic ketoacidosis](#_Toc808)**

**[7.2 Sensitive analysis of lower limb amputations](#_Toc8747)**

**[7.3 Sensitive analysis of urinary tract infections](#_Toc26383)**

**[7.4 Sensitive analysis of genital tract infections](#_Toc5897)**

**[7.5 Sensitive analysis of bone fracture](#_Toc7644)**

**[7.6 Sensitive analysis of hypoglycemia](#_Toc19726)**

**[Appendix 8: Confunnel plots](#_Toc7332)**

**[8.1 Confunnel plots for diabetic ketoacidosis](#_Toc24017)**

**[8.2 Confunnel plots for lower limb amputations](#_Toc29742)**

**[8.3 Confunnel plots for urinary tract infections](#_Toc9818)**

**[8.4 Confunnel plots for genital tract infections](#_Toc26427)**

**[8.5 Confunnel plots for bone fracture](#_Toc14555)**

**[8.6 Confunnel plots for hypoglycemia](#_Toc30687)**

**[Appendix 9: Results of subgroup analysis by study region](#_Toc3356)**

**[Appendix 1: PRISMA checklist](#_Toc15270)**

| **Section and Topic** | **Item #** | **Checklist item** | **Location where item is reported** |  |
| --- | --- | --- | --- | --- |
| **TITLE** | | |  | |
| Title | 1 | Identify the report as a systematic review. | 1 | |
| **ABSTRACT** | | |  | |
| Abstract | 2 | See the PRISMA 2020 for Abstracts checklist. | 2 | |
| **INTRODUCTION** | | |  | |
| Rationale | 3 | Describe the rationale for the review in the context of existing knowledge. | 3 | |
| Objectives | 4 | Provide an explicit statement of the objective(s) or question(s) the review addresses. | 3,4 | |
| **METHODS** | | |  | |
| Eligibility criteria | 5 | Specify the inclusion and exclusion criteria for the review and how studies were grouped for the syntheses. | 4 | |
| Information sources | 6 | Specify all databases, registers, websites, organisations, reference lists and other sources searched or consulted to identify studies. Specify the date when each source was last searched or consulted. | 4 | |
| Search strategy | 7 | Present the full search strategies for all databases, registers and websites, including any filters and limits used. | 4,5 | |
| Selection process | 8 | Specify the methods used to decide whether a study met the inclusion criteria of the review, including how many reviewers screened each record and each report retrieved, whether they worked independently, and if applicable, details of automation tools used in the process. | 5 | |
| Data collection process | 9 | Specify the methods used to collect data from reports, including how many reviewers collected data from each report, whether they worked independently, any processes for obtaining or confirming data from study investigators, and if applicable, details of automation tools used in the process. | 5 | |
| Data items | 10a | List and define all outcomes for which data were sought. Specify whether all results that were compatible with each outcome domain in each study were sought (e.g. for all measures, time points, analyses), and if not, the methods used to decide which results to collect. | 5 | |
|  | 10b | List and define all other variables for which data were sought (e.g. participant and intervention characteristics, funding sources). Describe any assumptions made about any missing or unclear information. | 5 | |
| Study risk of bias assessment | 11 | Specify the methods used to assess risk of bias in the included studies, including details of the tool(s) used, how many reviewers assessed each study and whether they worked independently, and if applicable, details of automation tools used in the process. | 5 | |
| Effect measures | 12 | Specify for each outcome the effect measure(s) (e.g. risk ratio, mean difference) used in the synthesis or presentation of results. | 6 | |
| Synthesis methods | 13a | Describe the processes used to decide which studies were eligible for each synthesis (e.g. tabulating the study intervention characteristics and comparing against the planned groups for each synthesis (item #5)). | 6 | |
|  | 13b | Describe any methods required to prepare the data for presentation or synthesis, such as handling of missing summary statistics, or data conversions. | 6 | |
|  | 13c | Describe any methods used to tabulate or visually display results of individual studies and syntheses. | 6 | |
|  | 13d | Describe any methods used to synthesize results and provide a rationale for the choice(s). If meta-analysis was performed, describe the model(s), method(s) to identify the presence and extent of statistical heterogeneity, and software package(s) used. | 6 | |
|  | 13e | Describe any methods used to explore possible causes of heterogeneity among study results (e.g. subgroup analysis, meta-regression). | 6 | |
|  | 13f | Describe any sensitivity analyses conducted to assess robustness of the synthesized results. | 6 | |
| Reporting bias assessment | 14 | Describe any methods used to assess risk of bias due to missing results in a synthesis (arising from reporting biases). | 6 | |
| Certainty assessment | 15 | Describe any methods used to assess certainty (or confidence) in the body of evidence for an outcome. | 6 | |
| **RESULTS** | | |  | |
| Study selection | 16a | Describe the results of the search and selection process, from the number of records identified in the search to the number of studies included in the review, ideally using a flow diagram. | 7 | |
|  | 16b | Cite studies that might appear to meet the inclusion criteria, but which were excluded, and explain why they were excluded. | 7 | |
| Study characteristics | 17 | Cite each included study and present its characteristics. | 7 | |
| Risk of bias in studies | 18 | Present assessments of risk of bias for each included study. | 10 | |
| Results of individual studies | 19 | For all outcomes, present, for each study: (a) summary statistics for each group (where appropriate) and (b) an effect estimate and its precision (e.g. confidence/credible interval), ideally using structured tables or plots. | 7,8 | |
| Results of syntheses | 20a | For each synthesis, briefly summarise the characteristics and risk of bias among contributing studies. | 7-10 | |
|  | 20b | Present results of all statistical syntheses conducted. If meta-analysis was done, present for each the summary estimate and its precision (e.g. confidence/credible interval) and measures of statistical heterogeneity. If comparing groups, describe the direction of the effect. | 7-10 | |
|  | 20c | Present results of all investigations of possible causes of heterogeneity among study results. | 7-10 | |
|  | 20d | Present results of all sensitivity analyses conducted to assess the robustness of the synthesized results. | 7-10 | |
| Reporting biases | 21 | Present assessments of risk of bias due to missing results (arising from reporting biases) for each synthesis assessed. | 10 | |
| Certainty of evidence | 22 | Present assessments of certainty (or confidence) in the body of evidence for each outcome assessed. | 10 | |
| **DISCUSSION** | | |  | |
| Discussion | 23a | Provide a general interpretation of the results in the context of other evidence. | 10-13 | |
|  | 23b | Discuss any limitations of the evidence included in the review. | 13,14 | |
|  | 23c | Discuss any limitations of the review processes used. | 13,14 | |
|  | 23d | Discuss implications of the results for practice, policy, and future research. | 13,14 | |
| **OTHER INFORMATION** | | |  | |
| Registration and protocol | 24a | Provide registration information for the review, including register name and registration number, or state that the review was not registered. | 4 | |
|  | 24b | Indicate where the review protocol can be accessed, or state that a protocol was not prepared. | 4 | |
|  | 24c | Describe and explain any amendments to information provided at registration or in the protocol. | 4 | |
| Support | 25 | Describe sources of financial or non-financial support for the review, and the role of the funders or sponsors in the review. | 15 | |
| Competing interests | 26 | Declare any competing interests of review authors. | 15 | |
| Availability of data, code and other materials | 27 | Report which of the following are publicly available and where they can be found: template data collection forms; data extracted from included studies; data used for all analyses; analytic code; any other materials used in the review. | 15 | |

**Appendix 2. [Search](C:/Users/Administrator/AppData/Local/youdao/dict/Application/8.9.9.0/resultui/html/index.html#/javascript:;) [strategy](C:/Users/Administrator/AppData/Local/youdao/dict/Application/8.9.9.0/resultui/html/index.html#/javascript:;)**

**Take Pubmed for example**

| **SGLT-2i** | sodium-dependent glucose co-transporter 2 inhibitors, sodium–glucose co-transporter 2 inhibitors, sodium/glucose cotransporter 2 inhibitors, [sodium–glucose cotransporter 2 inhibitors](https://www.ncbi.nlm.nih.gov/pmc/articles/PMC5850151/), sodium-glucose transporter 2 inhibitors, sodium glucose transporter 2 inhibitors, sodium-glucose transporter 2 inhibitor, sodium glucose transporter 2 inhibitor, SGLT 2 inhibitors, gliflozins, SGLT2 inhibitors, gliflozin, SGLT-2 inhibitor, SGLT 2 inhibitor, SGLT2 inhibitor, SGLT-2 inhibitors, Ertugliflozin, Dapagliflozin, Canagliflozin, Empagliflozin, Ipragliflozin, Tofogliflozin, Luseogliflozin, ASP1941, Remogliflozin etabonate, LX4211, Bexagliflozin, Gliflozins, Henagliflozin, Rongliflozin, ISIS 388626, BMS-512148 |
| --- | --- |
| ****DKA**** | **diabetic ketosis, diabetic ketoacidosis**, d**iabetic acidosis, ketoacidosis, diabetic ketoacidoses, ketoacidoses, ketosis, acidosis, acidoses, diabetic acidoses, diabetic ketosis, diabetic ketoses, DKA, diabetes mellitus ketoacidosis** |
| ****LLA**** | l**ower limb amputation, lower extremity amputation, amputation**, a**mputations, amputate, BKLE, BKA** |
| ****UTIs**** | **urinary infection, urinary tract infection, urinary system infection, UTI, UTIs, u**rinary tract infections |
| ****GTIs**** | RTI, RTIs, reproductive tract infection, genital tract infection,GTI, GTIs, reproductive tract infections, genital tract infections |
| **[bone fracture](C:/Users/Administrator/AppData/Local/youdao/dict/Application/8.9.6.0/resultui/html/index.html#/javascript:;)** | [fracture](C:/Users/Administrator/AppData/Local/youdao/dict/Application/8.9.6.0/resultui/html/index.html#/javascript:;), [bone fracture](C:/Users/Administrator/AppData/Local/youdao/dict/Application/8.9.6.0/resultui/html/index.html#/javascript:;) |
| ****hypoglycemia**** | **severe hypoglycemia, hypoglycemia** |
| **safety** | safety, safeties |
| ****adverse effects**** | **adverse effects**, **side effects, adverse reaction, adverse drug reaction, ADR, severe adverse reaction** |
| ****RCT**** | randomized controlled trial, randomized control trial, RCTs and RCT |

****Logical relations:**** SGLT-2i and (DKA or LLA or UTIs or GTIs or [bone fracture](C:/Users/Administrator/AppData/Local/youdao/dict/Application/8.9.6.0/resultui/html/index.html#/javascript:;) or hypoglycemia or safety or adverse effects) not **RCT**

**Appendix 3. Risk of Bias Assessment**

| **Study** | **Selection** | | | | **Comparability** | | **Outcome** | | | **In total** |
| --- | --- | --- | --- | --- | --- | --- | --- | --- | --- | --- |
|  | 1* | 2* | 3* | 4* | 5* | 6* | 7* | 8* | 9* |  |
| Patorno E 2021 | 1 | 1 | 1 | 0 | 1 | 1 | 1 | 1 | 1 | 8 |
| Han SJ 2021 | 1 | 1 | 1 | 0 | 1 | 1 | 1 | 1 | 1 | 8 |
| Fralick M 2021 | 1 | 1 | 1 | 1 | 1 | 1 | 1 | 1 | 1 | 9 |
| Yu OHY 2020 | 1 | 1 | 1 | 1 | 1 | 1 | 1 | 1 | 1 | 9 |
| Udell JA 2020 | 1 | 1 | 1 | 1 | 1 | 1 | 1 | 1 | 1 | 9 |
| Lee HF 2020 | 1 | 1 | 1 | 1 | 1 | 1 | 1 | 1 | 1 | 9 |
| Fralick M 2020 | 1 | 1 | 1 | 1 | 1 | 1 | 1 | 1 | 1 | 9 |
| Douros A 2020 | 1 | 1 | 1 | 1 | 1 | 1 | 1 | 1 | 1 | 9 |
| Yang JY 2019 | 1 | 1 | 1 | 1 | 1 | 1 | 1 | 1 | 1 | 9 |
| Wang L 2019 | 1 | 1 | 1 | 1 | 1 | 1 | 1 | 1 | 1 | 9 |
| Fralick M 2019 | 1 | 1 | 1 | 1 | 1 | 1 | 1 | 1 | 1 | 9 |
| Dawwas GK 2019 | 1 | 1 | 1 | 1 | 1 | 1 | 1 | 1 | 1 | 9 |
| Dave CV 2019 | 1 | 1 | 1 | 0 | 1 | 1 | 1 | 1 | 1 | 8 |
| Dave CV 2019 | 1 | 1 | 1 | 0 | 1 | 1 | 1 | 1 | 1 | 8 |
| Adimadhyam S 2019 | 1 | 1 | 1 | 1 | 1 | 1 | 1 | 1 | 1 | 9 |
| Yuan Z 2018 | 1 | 1 | 1 | 1 | 1 | 1 | 1 | 1 | 1 | 9 |
| Udell JA 2018 | 1 | 1 | 1 | 1 | 1 | 1 | 1 | 1 | 1 | 9 |
| Toulis KA 2018 | 1 | 1 | 1 | 1 | 1 | 0 | 1 | 1 | 1 | 8 |
| Persson F 2018 | 1 | 1 | 1 | 1 | 1 | 1 | 1 | 1 | 1 | 9 |
| Kim YG 2018 | 1 | 1 | 1 | 1 | 1 | 1 | 1 | 1 | 1 | 9 |
| Chang HY 2018 | 1 | 1 | 1 | 1 | 1 | 1 | 1 | 1 | 1 | 9 |
| Wang Y 2017 | 1 | 1 | 1 | 1 | 1 | 1 | 1 | 1 | 1 | 9 |
| Birkeland KI 2017 | 1 | 1 | 1 | 1 | 1 | 1 | 1 | 1 | 1 | 9 |
| Norhammar A 2018 | 1 | 1 | 1 | 0 | 1 | 1 | 1 | 1 | 1 | 8 |
| Nyström T 2017 | 1 | 1 | 1 | 0 | 1 | 1 | 1 | 1 | 1 | 8 |
| Pasternak B 2019 | 1 | 1 | 1 | 1 | 1 | 1 | 1 | 1 | 1 | 9 |
| Ryan PB 2018 | 1 | 1 | 1 | 0 | 1 | 1 | 1 | 1 | 1 | 8 |
| Dawwas GK 2022 | 1 | 1 | 1 | 1 | 1 | 0 | 1 | 1 | 1 | 8 |
| Fralick M 2021 | 1 | 1 | 1 | 1 | 1 | 0 | 1 | 1 | 1 | 8 |
| Ha KH 2022 | 1 | 1 | 1 | 1 | 1 | 1 | 1 | 1 | 1 | 9 |
| Laursen HVB 2021 | 1 | 1 | 1 | 1 | 1 | 0 | 1 | 0 | 1 | 7 |
| Paul SK 2021 | 1 | 1 | 1 | 1 | 1 | 1 | 1 | 1 | 1 | 9 |
| Werkman NCC 2021 | 1 | 1 | 1 | 1 | 1 | 1 | 1 | 1 | 1 | 9 |
| Zerovnik S 2022 | 1 | 1 | 1 | 1 | 1 | 0 | 1 | 1 | 1 | 8 |
| Patorno E 2022 | 1 | 1 | 1 | 1 | 1 | 1 | 1 | 0 | 1 | 8 |
| Al-Mashhadi ZK 2022 | 1 | 1 | 1 | 1 | 1 | 1 | 1 | 1 | 1 | 9 |
| Dalem JV 2022 | 1 | 1 | 1 | 1 | 1 | 1 | 1 | 1 | 1 | 9 |
| D'Andrea E 2023 | 1 | 1 | 1 | 1 | 1 | 1 | 1 | 1 | 1 | 9 |
| Fu EL 2023 | 1 | 1 | 1 | 1 | 1 | 1 | 1 | 1 | 1 | 9 |
| Htoo PT 2023 | 1 | 1 | 1 | 0 | 1 | 1 | 1 | 1 | 1 | 8 |

**Appendix 4: The events and incidence rate of outcomes**

**4.1 The events and incidence rate of diabetic ketoacidosis**

| **Study** | **Observation group** | | | **Control group** | | |
| --- | --- | --- | --- | --- | --- | --- |
|  | **Events** | **Incidence rate/1,000 person-years** | **Sample size** | **Events** | **Incidence rate/1,000 person-years** | **Sample size** |
| **Total group (SGLT-2i vs oGLD)** | | | | | | |
| Patorno E 2021(SGLT-2i vs GLP-1RA) | 79 | 2.37 | 45047 | 50 | 1.65 | 45047 |
| Fralick M 2021(SGLT-2i vs DPP-4i) | 68 | 2.2 | 29916 | 43 | 1.2 | 29916 |
| Douros A 2020 (SGLT-2i vs DPP-4i) | 372 | 2.03 | 202186 | 133 | 0.75 | 202186 |
| Wang L 2019 (SGLT-2i vs SU) | 249 | —— | 131542 | 185 | —— | 131542 |
| Wang L 2019 (SGLT-2i vs DPP-4i) | 267 | —— | 149775 | 253 | —— | 149775 |
| Wang L 2019 (SGLT-2i vs GLP-1RA) | 242 | —— | 123792 | 213 | —— | 123792 |
| Wang L 2019 (SGLT-2i vs TZD) | 108 | —— | 62084 | 120 | —— | 62084 |
| Wang L 2019 (SGLT-2i vs insulin) | 180 | —— | 92890 | 230 | —— | 92890 |
| Wang L 2019 (SGLT-2i vs metformin) | 150 | —— | 73994 | 120 | —— | 73994 |
| Kim YG 2018 (SGLT-2i vs DPP-4i) | 29 | 0.614 | 56325 | 34 | 0.686 | 56325 |
| Wang Y 2017 (SGLT-2i vs oGLD) | 16 | —— | 27515 | 11 | —— | 27515 |
| Pasternak B 2019 (SGLT-2i vs DPP-4i) | 27 | 1.4 | 20983 | 16 | 0.6 | 20983 |
| Dawwas GK 2022 (SGLT-2i vs DPP-4i) | 343 | 6.0 | 85125 | 256 | 4.3 | 85125 |
| Dawwas GK 2022 (SGLT-2i vs SU) | 313 | 6.3 | 72436 | 227 | 4.5 | 72436 |
| Fralick M 2021 (SGLT-2i vs metformin) | 32 | 4.25 | 9964 | 23 | 2.32 | 9964 |
| Laursen HVB 2021 (SGLT-2i vs GLP-1RA) | 13 | 0.84 | 10923 | 27 | 0.53 | 10923 |
| Patorno E 2022 (SGLT-2i vs DPP-4i) | 52 | 2.72 | 39072 | 28 | 1.55 | 39072 |
| D'Andrea E 2023(SGLT-2i vs DPP-4i) | 66 | 2.25 | 43637 | 36 | 1.27 | 43637 |
| Fu EL 2023 (SGLT-2i vs GLP-1RA) | 59 | 3.4 | 28847 | 58 | 3.1 | 28847 |
| **Total** | 2665 | —— | 1233569 | 2063 | —— | 1306053 |
| **Mean** | —— | 2.86 | —— | —— | 1.87 | —— |
| **Subgroup (SGLT-2i vs GLP-1RA)** | | | | | | |
| Patorno E 2021(SGLT-2i vs GLP-1RA) | 79 | 2.37 | 45047 | 50 | 1.65 | 45047 |
| Wang L 2019 (SGLT-2i vs GLP-1RA) | 242 | —— | 123792 | 213 | —— | 123792 |
| Laursen HVB 2021 (SGLT-2i vs GLP-1RA) | 13 | 0.84 | 10923 | 27 | 0.53 | 10923 |
| Fu EL 2023 (SGLT-2i vs GLP-1RA) | 59 | 3.4 | 28847 | 58 | 3.1 | 28847 |
| **Total** | 393 | —— | 208609 | 348 | —— | 179762 |
| **Mean** | —— | 2.20 | —— | —— | 1.76 | —— |
| **Subgroup (SGLT-2i vs DPP-4i)** | | | | | | |
| Fralick M 2021(SGLT-2i vs DPP-4i) | 68 | 2.2 | 29916 | 43 | 1.2 | 29916 |
| Douros A 2020 (SGLT-2i vs DPP-4i) | 372 | 2.03 | 202186 | 133 | 0.75 | 202186 |
| Wang L 2019 (SGLT-2i vs DPP-4i) | 267 | —— | 149775 | 253 | —— | 149775 |
| Kim YG 2018 (SGLT-2i vs DPP-4i) | 29 | 0.614 | 56325 | 34 | 0.686 | 56325 |
| Pasternak B 2019 (SGLT-2i vs DPP-4i) | 27 | 1.4 | 20983 | 16 | 0.6 | 20983 |
| Dawwas GK 2022 (SGLT-2i vs DPP-4i) | 343 | 6 | 85125 | 256 | 4.3 | 85125 |
| Patorno E 2022 (SGLT-2i vs DPP-4i) | 52 | 2.72 | 39072 | 28 | 1.55 | 39072 |
| **study** | **Observation group** | | | **Control group** | | |
|  | **Events** | **Incidence rate/1,000 person-years** | **Sample size** | **Events** | **Incidence rate/1,000 person-years** | **Sample size** |
| D'Andrea E 2023(SGLT-2i vs DPP-4i) | 66 | 2.25 | 43637 | 36 | 1.27 | 43637 |
| **Total** | 1224 | —— | 627019 | 799 | —— | 627019 |
| **Mean** | —— | 2.46 | —— | —— | 1.48 | —— |
| **Subgroup (Canagliflozin vs DPP-4i)** | | | | | | |
| Douros A 2020 (Canagliflozin vs DPP-4i) | 200 | 2.25 | 78779 | 58 | 0.67 | 78779 |
| Wang L 2019 (Canagliflozin vs DPP-4i) | 218 | —— | 100921 | 171 | —— | 100921 |
| Dawwas GK 2022 (Canagliflozin vs DPP-4i) | 235 | 7.6 | 44486 | 147 | 4.4 | 44486 |
| **Total** | 653 | —— | 224186 | 376 | —— | 224186 |
| **Mean** | —— | 4.93 | —— | —— | 2.54 | —— |
| **Subgroup (Dapagliflozin vs DPP-4i)** | | | | | | |
| Douros A 2020 (Dapagliflozin vs DPP-4i) | 58 | 2.03 | 36746 | 32 | 1.18 | 36746 |
| Wang L 2019 (Dapagliflozin vs DPP-4i) | 72 | —— | 49617 | 67 | —— | 49617 |
| Dawwas GK 2022 (Dapagliflozin vs DPP-4i) | 41 | 8.3 | 9714 | 18 | 2.7 | 9714 |
| **Total** | 171 | —— | 96077 | 117 | —— | 96077 |
| **Mean** | —— | 5.17 | —— | —— | 1.94 | —— |
| **Subgroup (Empagliflozin vs DPP-4i)** | | | | | | |
| Douros A 2020 (Empagliflozin vs DPP-4i) | 25 | 1.47 | 26728 | 11 | 0.63 | 26728 |
| Wang L 2019 (Empagliflozin vs DPP-4i) | 62 | —— | 50480 | 57 | —— | 50480 |
| Dawwas GK 2022 (Empagliflozin vs DPP-4i) | 102 | 5.3 | 35345 | 96 | 3.9 | 35345 |
| Patorno E 2022 (Empagliflozin vs DPP-4i) | 52 | 2.72 | 39072 | 28 | 1.55 | 39072 |
| **Total** | 241 | —— | 151625 | 192 | —— | 151625 |
| **Mean** | —— | 3.16 | —— | —— | 2.03 | —— |

Legend: SGLT-2i = sodium-glucose cotransporter 2 inhibitors, oGLD = other glucose lowering drugs, GLP-1RA = GLP-1 receptor agonist, DPP-4i = dipeptidyl peptidase 4 inhibitors

**4.2 The events and incidence rate of lower limb amputation**

| **Study** | **Observation group** | | | **Control group** | | |
| --- | --- | --- | --- | --- | --- | --- |
|  | **Events** | **Incidence rate/1,000 person-years** | **Sample size** | **Events** | **Incidence rate/1,000 person-years** | **Sample size** |
| **Total group (SGLT-2i vs oGLD)** | | | | | | |
| Patorno E 2021(SGLT-2i vs GLP-1RA) | 104 | 3.12 | 45047 | 67 | 2.22 | 45047 |
| Yu OHY 2020 (SGLT-2i vs DPP-4i) | 253 | 1.3 | 207817 | 281 | 1.5 | 207817 |
| Fralick M 2020 (Canagliflozin vs GLP-1RA) | 417 | —— | 155420 | 294 | —— | 155420 |
| Yang JY 2019 (SGLT-2i vs DPP-4i) | 70 | 2.3 | 50189 | 136 | 1.6 | 50189 |
| Yang JY 2019 (SGLT-2i vs SU) | 56 | 1.9 | 48954 | 207 | 1.9 | 48954 |
| Dawwas GK 2019 (SGLT-2i vs SU) | 92 | 1.5 | 62767 | 158 | 1.9 | 62767 |
| Dawwas GK 2019 (SGLT-2i vs DPP-4i) | 120 | 1.8 | 66633 | 171 | 1.9 | 66633 |
| Yuan Z 2018 (Canagliflozin vs oGLD) | 99 | 1.18 | 63845 | 87 | 1.12 | 63845 |
| Chang HY 2018 | 18 |  | 39869 | 283 |  | 914037 |
| Pasternak B 2019 (SGLT-2i vs DPP-4i) | 59 | 3.1 | 20983 | 64 | 2.6 | 20983 |
| Ryan PB 2018(Canagliflozin vs oGLD) | 295 | —— | 111332 | 1308 | —— | 445367 |
| Ryan PB 2018(Empag or Dapag vs oGLD) | 150 | —— | 78248 | 829 | —— | 324755 |
| Werkman NCC 2021(SGLT-2i vs SU) | 16 | 0.97 | 10927 | 50 | 1.29 | 19651 |
| Zerovnik S 2022(SGLT-2i vs DPP-4i) | 37 | 4.3 | 2939 | 25 | 2.3 | 2939 |
| Patorno E 2022 (SGLT-2i vs DPP-4i) | 53 | 2.78 | 39072 | 44 | 2.43 | 39072 |
| Paul SK 2021(SGLT-2i vs GLP-1RA) |  | 1.26 | 169739 |  | 1.23 | 149826 |
| Paul SK 2021(SGLT-2i vs DPP-4i) |  | 1.26 | 169739 |  | 1.42 | 448225 |
| Paul SK 2021(SGLT-2i vs oGLD) |  | 1.26 | 169739 |  | 2.03 | 1954353 |
| D'Andrea E 2023(SGLT-2i vs DPP-4i) | 78 | 2.56 | 43637 | 68 | 2.46 | 43637 |
| Fu EL 2023 (SGLT-2i vs GLP-1RA) | 108 | 6.2 | 28847 | 70 | 3.7 | 28847 |
| **Total** | 2025 | —— | 1585743 | 4142 | —— | 5092364 |
| **Mean** | —— | 2.30 | —— | —— | 1.98 | —— |
| **Subgroup (SGLT-2i vs DPP-4i)** | | | | | | |
| Yu OHY 2020 (SGLT-2i vs DPP-4i) | 253 | 1.3 | 207817 | 281 | 1.5 | 207817 |
| Yang JY 2019 (SGLT-2i vs DPP-4i) | 70 | 2.3 | 50189 | 136 | 1.6 | 50189 |
| Dawwas GK 2019 (SGLT-2i vs DPP-4i) | 120 | 1.8 | 66633 | 171 | 1.9 | 66633 |
| Chang HY 2018 (SGLT-2i vs DPP-4i) |  | 10.53 | 39869 | 41 | 8.52 | 105023 |
| Pasternak B 2019 (SGLT-2i vs DPP-4i) | 59 | 3.1 | 20983 | 64 | 2.6 | 20983 |
| Paul SK 2021(SGLT-2i vs DPP-4i) |  | 1.26 | 169 739 |  | 1.42 | 448 225 |
| Zerovnik S 2022(SGLT-2i vs DPP-4i) | 37 | 4.3 | 2939 | 25 | 2.3 | 2939 |
| Patorno E 2022 (SGLT-2i vs DPP-4i) | 53 | 2.78 | 39072 | 44 | 2.43 | 39072 |
| D'Andrea E 2023(SGLT-2i vs DPP-4i) | 78 | 2.56 | 43637 | 68 | 2.46 | 43637 |
| **Total** | 670 | —— | 471139 | 830 | —— | 536293 |
| **Mean** | —— | 3.33 | —— | —— | 2.75 | —— |
| **Subgroup (SGLT-2i vs GLP-1RA)** | | | | | | |
| Patorno E 2021(SGLT-2i vs GLP-1RA) | 104 | 3.12 | 45047 | 67 | 2.22 | 45047 |
| Fralick M 2020 (Canagliflozin vs GLP-1RA) | 417 | —— | 155420 | 294 | —— | 155420 |
| Chang HY 2018 (SGLT-2i vs GLP-1RA) |  | 10.53 | 39869.00 | 11 | 7.10 | 39120.00 |
| Paul SK 2021(SGLT-2i vs GLP-1RA) |  | 1.26 | 169 739 |  | 1.23 | 149826 |
| Fu EL 2023 (SGLT-2i vs GLP-1RA) | 108 | 6.2 | 28847 | 70 | 3.7 | 28847 |
| **Total** | 629 | —— | 269183 | 442 | —— | 418260 |
| **Mean** |  | 5.28 |  |  | 3.56 |  |
| **Subgroup (Canagliflozin vs SU)** | | | | | | |
| Yang JY 2019 (SGLT-2i vs SU) | 56 | 1.9 | 48954 | 207 | 1.9 | 48954 |
| Dawwas GK 2019 (SGLT-2i vs SU) | 92 | 1.5 | 62767 | 158 | 1.9 | 62767 |
| Werkman NCC 2021(SGLT-2i vs SU) | 16 | 0.97 | 10927 | 50 | 1.29 | 19651 |
| **Total** | 164 |  | 122648 | 415 |  | 131372 |
| **Mean** |  | 1.46 |  |  | 1.70 |  |
| **Subgroup (SGLT-2i vs oGLD CVD(+))** | | | | | | |
| Fralick M 2020 (Canag vs GLP-1RA)Age<65 | 87 | 9.74 | 10763 | 69 | 8.57 | 10763 |
| Fralick M 2020 (Canag vs GLP-1RA)Age≥65 | 132 | 9.32 | 19495 | 74 | 5.46 | 19495 |
| Ryan PB 2018(Canagliflozin vs oGLD) | 295 | —— | 111332 | 1308 | —— | 445367 |
| Ryan PB 2018(Empag or Dapag vs oGLD) | 150 | —— | 78248 | 829 | —— | 324755 |
| Fu EL 2023 (SGLT-2i vs GLP-1RA) | 108 | 6.2 | 28847 | 70 | 3.7 | 28847 |
| **Total** | 772 |  | 248685 | 2350 |  | 829227 |
| **Mean** |  | 8.42 |  |  | 5.91 |  |
| **Subgroup (SGLT-2i vs oGLD CVD(-))** | | | | | | |
| Fralick M 2020 (Canag vs GLP-1RA)Age<65 | 117 | 1.67 | 80640 | 98 | 1.55 | 80640 |
| Fralick M 2020 (Canag vs GLP-1RA)Age≥65 | 81 | 2.34 | 44522 | 53 | 1.61 | 44522 |
| **Total** | 198 |  | 125162 | 151 |  | 125162 |
| **Mean** |  | 2.01 |  |  | 1.58 |  |
| **Subgroup (SGLT-2i vs oGLD PVD＞10%)** | | | | | | |
| Patorno E 2021(SGLT-2i vs GLP-1RA) | 104 | 3.12 | 45047 | 67 | 2.22 | 45047 |
| Fralick M 2020 (Canagvs GLP-1RA)Age<65 | 87 | 9.74 | 10763 | 69 | 8.57 | 10763 |
| Fralick M 2020 (Canagvs GLP-1RA)Age≥65 | 132 | 9.32 | 19495 | 74 | 5.46 | 19495 |
| Ryan PB 2018(Canagliflozin vs oGLD) | 295 | —— | 111332 | 1308 | —— | 445367 |
| Ryan PB 2018(Empag or Dapag vs oGLD) | 150 | —— | 78248 | 829 | —— | 324755 |
| Fu EL 2023 (SGLT-2i vs GLP-1RA) | 108 | 6.2 | 28847 | 70 | 3.7 | 28847 |
| **Total** | 876 |  | 293732 | 2417 |  | 874274 |
| **Mean** |  | 7.10 |  |  | 4.99 |  |
| **Subgroup (SGLT-2i vs oGLD PVD＜10%)** | | | | | | |
| Yu OHY 2020 (SGLT-2i vs DPP-4i) | 253 | 1.3 | 207817 | 281 | 1.5 | 207817 |
| Fralick M 2020 (Canag vs GLP-1RA)Age<65 | 117 | 1.67 | 80640 | 98 | 1.55 | 80640 |
| Fralick M 2020 (Canag vs GLP-1RA)Age≥65 | 81 | 2.34 | 44522 | 53 | 1.61 | 44522 |
| Yang JY 2019 (SGLT-2i vs DPP-4i) | 70 | 2.3 | 50189 | 136 | 1.6 | 50189 |
| Yang JY 2019 (SGLT-2i vs SU) | 56 | 1.9 | 48954 | 207 | 1.9 | 48954 |
| Yuan Z 2018 (Canagliflozin vs oGLD) | 99 | 1.18 | 63845 | 87 | 1.12 | 63845 |
| Ryan PB 2018(Canagliflozin vs oGLD) | 295 | —— | 111332 | 1308 | —— | 445367 |
| Ryan PB 2018(Empag or Dapag vs oGLD) | 150 | —— | 78248 | 829 | —— | 324755 |
| Paul SK 2021(SGLT-2i vs GLP-1RA) |  | 1.26 | 169 739 |  | 1.23 | 149826 |
| Paul SK 2021(SGLT-2i vs DPP-4i) |  | 1.26 | 169 739 |  | 1.42 | 448 225 |
| Paul SK 2021(SGLT-2i vs oGLD) |  | 1.26 | 169 739 |  | 2.03 | 1954353 |
| Werkman NCC 2021(SGLT-2i vs SU) | 16 | 0.97 | 10927 | 50 | 1.29 | 19651 |
| Patorno E 2022 (SGLT-2i vs DPP-4i) | 53 | 2.78 | 39072 | 44 | 2.43 | 39072 |
| **Total** | 1190 |  | 735546 | 3093 |  | 1324812 |
| **Mean** |  | 1.66 |  |  | 1.61 |  |

Legend: SGLT-2i = sodium-glucose cotransporter 2 inhibitors, oGLD = other glucose lowering drugs, GLP-1RA = GLP-1 receptor agonist, DPP-4i = dipeptidyl peptidase 4 inhibitors, TZD = thiazolidinedione, SU = sulfonylureas, CVD = cardiovascular disease, PVD = peripheral vascular diseases

**4.3 The events and incidence rate of urinary tract infections**

| **Study** | **Observation group** | | | **Control group** | | |
| --- | --- | --- | --- | --- | --- | --- |
|  | **Events** | **Incidence rate/1,000 person-years** | **Sample size** | **Events** | **Incidence rate/1,000 person-years** | **Sample size** |
| **Total group (SGLT-2i vs oGLD)** | | | | | | |
| Patorno E 2021(SGLT-2i vs GLP-1RA) | 147 | 4.41 | 45047 | 161 | 5.33 | 45047 |
| Han SJ 2021(SGLT-2i vs DPP-4i) | —— | 20.37 | 15699 | —— | 19.17 | 15699 |
| Dave CV 2019 (SGLT-2i vs DPP-4i) | 61 | 1.76 | 16147 | 57 | 1.77 | 16147 |
| Dave CV 2019 (SGLT-2i vs GLP-1RA) | 73 | 2.15 | 14645 | 87 | 2.96 | 14645 |
| Fu EL 2023 (SGLT-2i vs GLP-1RA) | 334 | 19.2 | 28847 | 352 | 18.9 | 28847 |
| **Total** | 615 |  | 120385 | 305 |  | 120385 |
| **Mean** |  | 9.58 |  |  | 9.63 |  |

Legend: SGLT-2i = sodium-glucose cotransporter 2 inhibitors, oGLD = other glucose lowering drugs, GLP-1RA = GLP-1 receptor agonist, DPP-4i = dipeptidyl peptidase 4 inhibitors

**4.4 The events and incidence rate of genital tract infections**

| **Study** | **Observation group** | | | **Control group** | | |
| --- | --- | --- | --- | --- | --- | --- |
|  | **Events** | **Incidence rate/1,000 person-years** | **Sample size** | **Events** | **Incidence rate/1,000 person-years** | **Sample size** |
| **Total group (SGLT-2i vs oGLD)** | | | | | | |
| Patorno E 2021(SGLT-2i vs GLP-1RA) | 2623 | 82.31 | 45047 | 753 | 25.23 | 45047 |
| Han SJ 2021(SGLT-2i vs DPP-4i) | —— | 9.93 | 15699 | —— | 4.01 | 15699 |
| Dave CV 2019(SGLT-2i vs DPP-4i)F | 3599 | 135.5 | 129994 | 1247 | 48.5 | 129994 |
| Dave CV 2019(SGLT-2i vs DPP-4i)M | 684 | 19 | 156074 | 237 | 7.2 | 156074 |
| Fralick M 2021 (SGLT-2i vs metformin) | 282 | 38.31 | 9964 | 153 | 15.64 | 9964 |
| D'Andrea E 2023(SGLT-2i vs DPP-4i) | 1847 | 65.41 | 43637 | 780 | 27.99 | 43637 |
| Fu EL 2023 (SGLT-2i vs GLP-1RA) | 1018 | 60.2 | 28847 | 352 | 19 | 28847 |
| **Total** | 10178 |  | 343013 | 3616 |  | 343013 |
| **Mediam** |  | 58.67 |  |  | 21.08 |  |

Legend: SGLT-2i = sodium-glucose cotransporter 2 inhibitors, oGLD = other glucose lowering drugs, GLP-1RA = GLP-1 receptor agonist, DPP-4i = dipeptidyl peptidase 4 inhibitors

**4.5 The events and incidence rate of hypoglycemia**

| **Study** | **Observation group** | | | **Control group** | | |
| --- | --- | --- | --- | --- | --- | --- |
|  | **Events** | **Incidence rate/1,000 person-years** | **Sample size** | **Events** | **Incidence rate/1,000 person-years** | **Sample size** |
| **Total group (SGLT-2i vs oGLD)** | | | | | | |
| Han SJ 2021(SGLT-2i vs DPP-4i) | —— | 2.4 | 15699 | —— | 2.56 | 15699 |
| Fralick M 2021(SGLT-2i vs DPP-4i) | 71 | 2.3 | 29916 | 141 | 3.9 | 29916 |
| Norhammar A 2018(Dapagliflozin vs oGLD) | 209 | 18.7 | 7102 | 681 | 20.5 | 21306 |
| Nyström T 2017(Dapagliflozin vs insulin) |  | —— | 6139 |  | —— | 6139 |
| Fralick M 2021 (SGLT-2i vs metformin) | 22 | 2.92 | 9964 | 33 | 3.33 | 9964 |
| Fu EL 2023 (SGLT-2i vs GLP-1RA) | 325 | 18.7 | 28847 | 322 | 17.3 | 28847 |
| Htoo PT 2023 (SGLT-2i vs DPP-4i) | 692 | 9.8 | 82994 | 987 | 13 | 82994 |
| Htoo PT 2023 (SGLT-2i vs GLP-1RA) | 848 | 11.1 | 88726 | 898 | 12.5 | 88726 |
| **Total** | 2167 |  | 269387 | 3062 |  | 283591 |
| **Mean** |  | 9.42 |  |  | 10.44 |  |

Legend: SGLT-2i = sodium-glucose cotransporter 2 inhibitors, oGLD = other glucose lowering drugs, GLP-1RA = glucagon-like peptide-1 receptor agonist, DPP-4i = dipeptidyl peptidase 4 inhibitors

**Appendix 5: Forest plot for meta-analysis**

**5.1 Forest plot of SGLT-2i vs DPP-4i/GLP-1RA for diabetic ketoacidosis**


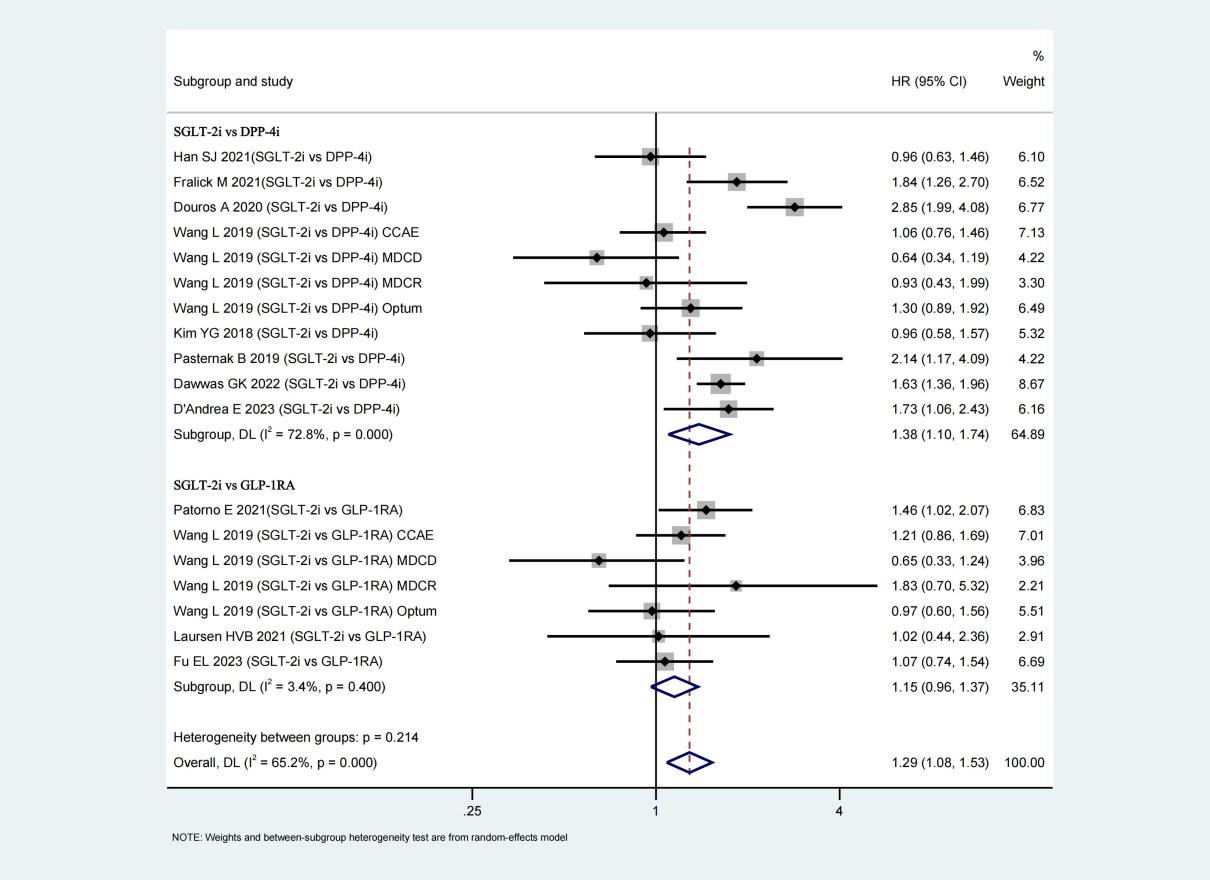


Legend: SGLT-2i = sodium-glucose cotransporter 2 inhibitors, GLP-1RA = glucagon-like peptide-1 receptor agonist, DPP-4i = dipeptidyl peptidase 4 inhibitors

**5.2 Forest plot of Canagliflozin/Dapagliflozin/Empagliflozin vs DPP-4i for diabetic ketoacidosis**


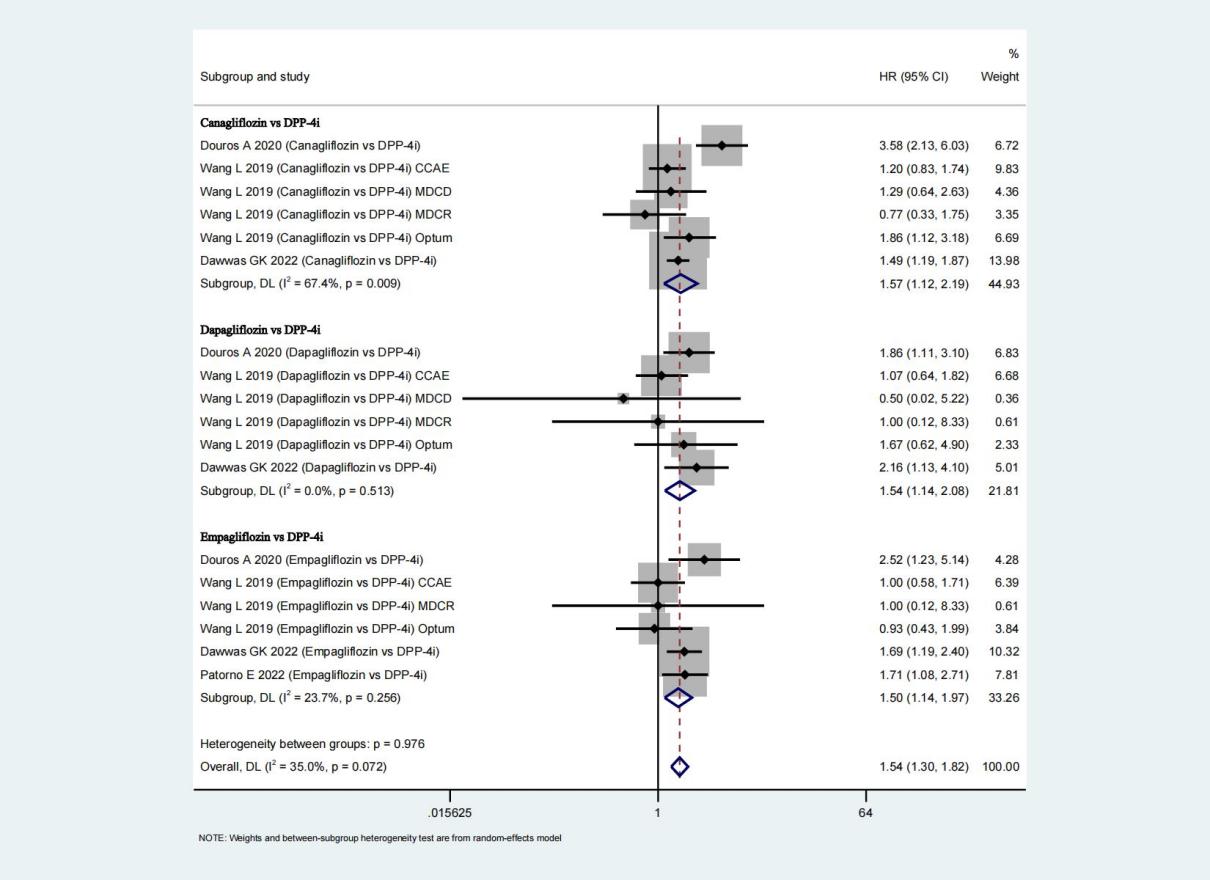


Legend: DPP-4i = dipeptidyl peptidase 4 inhibitors

**5.3 Forest plot of SGLT-2i vs DPP-4i/GLP-1RA/SU for lower limb amputations**


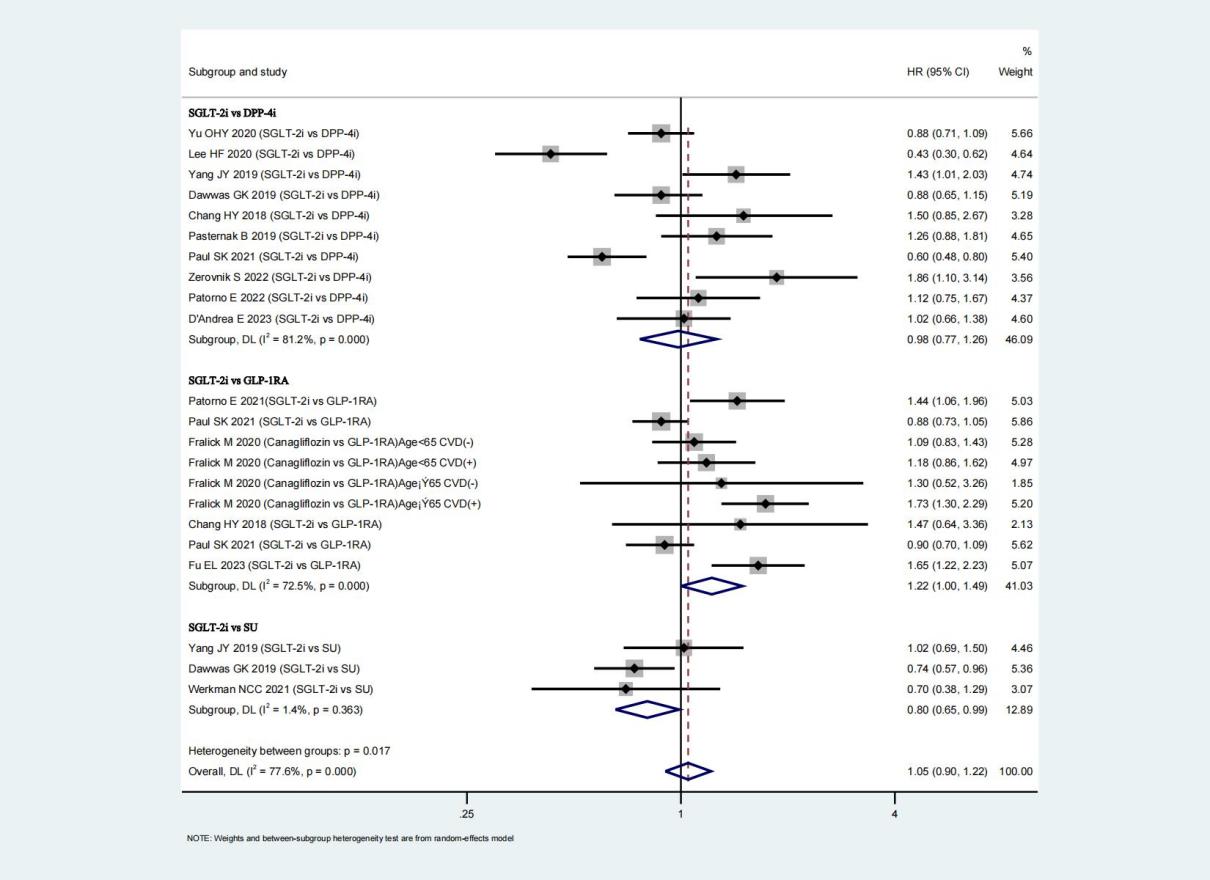


Legend: SGLT-2i = sodium-glucose cotransporter 2 inhibitors, GLP-1RA = glucagon-like peptide-1 receptor agonist, DPP-4i = dipeptidyl peptidase 4 inhibitors, SU = sulfonylureas

**5.4 Forest plot of Canagliflozin compared with other glucose-lowering drugs for lower limb amputations**


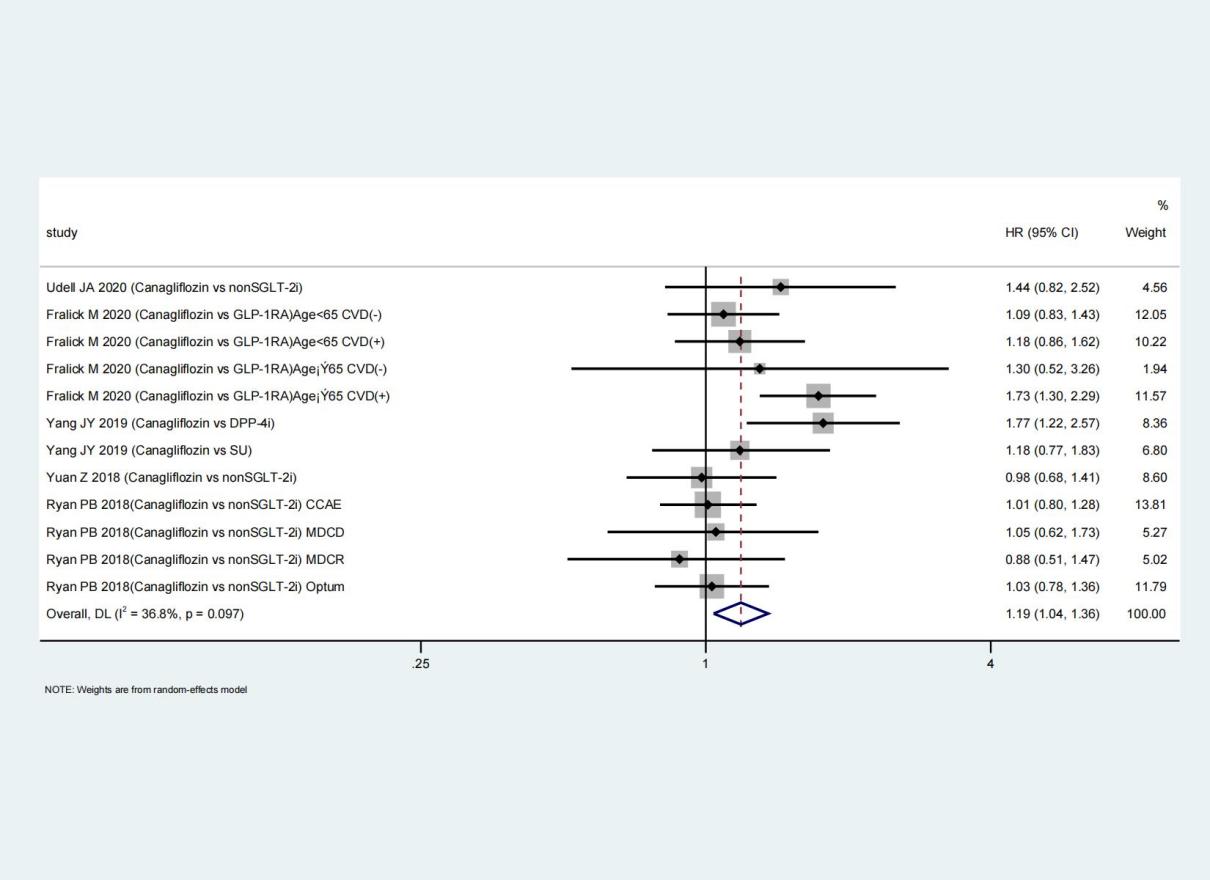


Legend: SGLT-2i = sodium-glucose cotransporter 2 inhibitors, GLP-1RA = glucagon-like peptide-1 receptor agonist, DPP-4i = dipeptidyl peptidase 4 inhibitors, SU = sulfonylureas, CVD = cardiovascular disease

**5.5 Forest plot for lower limb amputations according to the prevalence of peripheral vascular diseases**


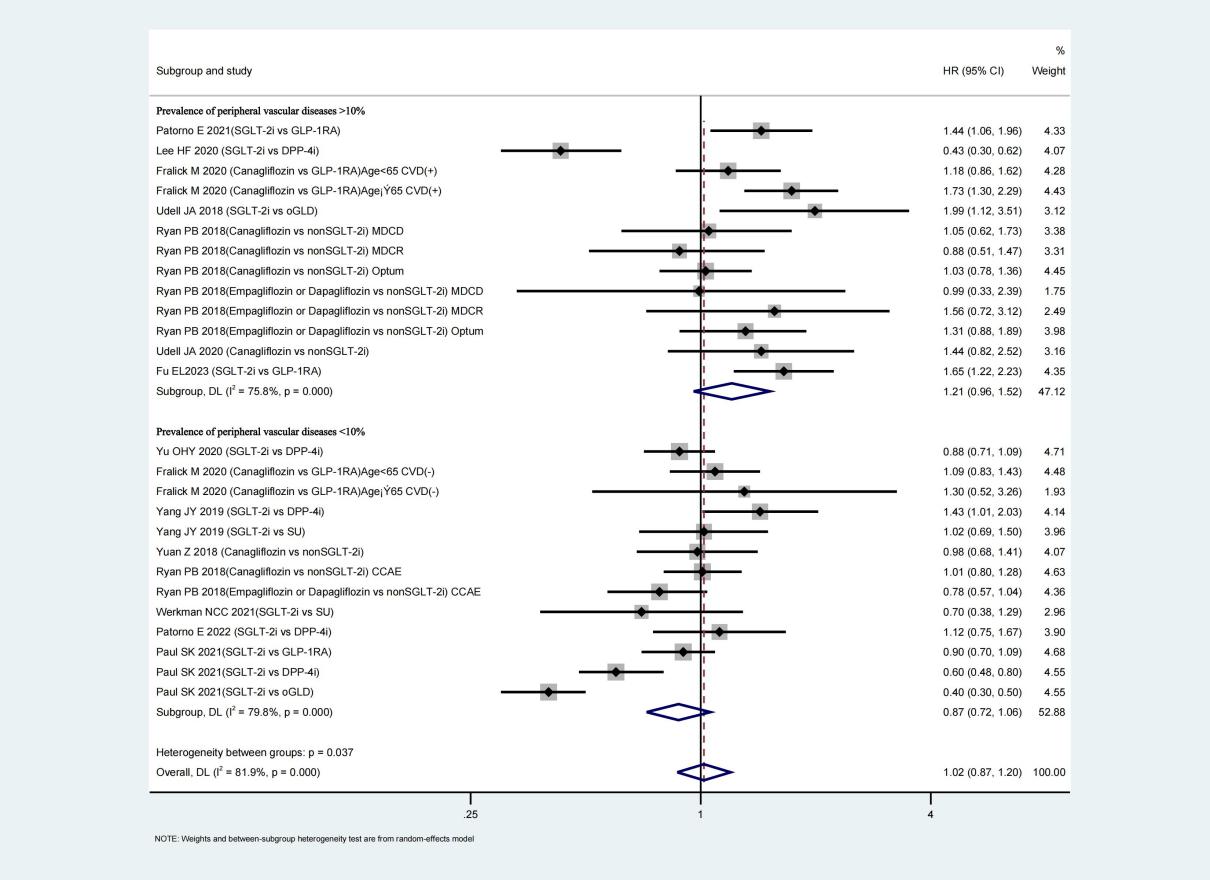


Legend: SGLT-2i = sodium-glucose cotransporter 2 inhibitors, oGLD = other glucose lowering drugs, GLP-1RA = glucagon-like peptide-1 receptor agonist, DPP-4i = dipeptidyl peptidase 4 inhibitors, SU = sulfonylureas, CVD = cardiovascular disease

**5.6 Forest plot for lower limb amputations in patients with or without prior cardiovascular disease**


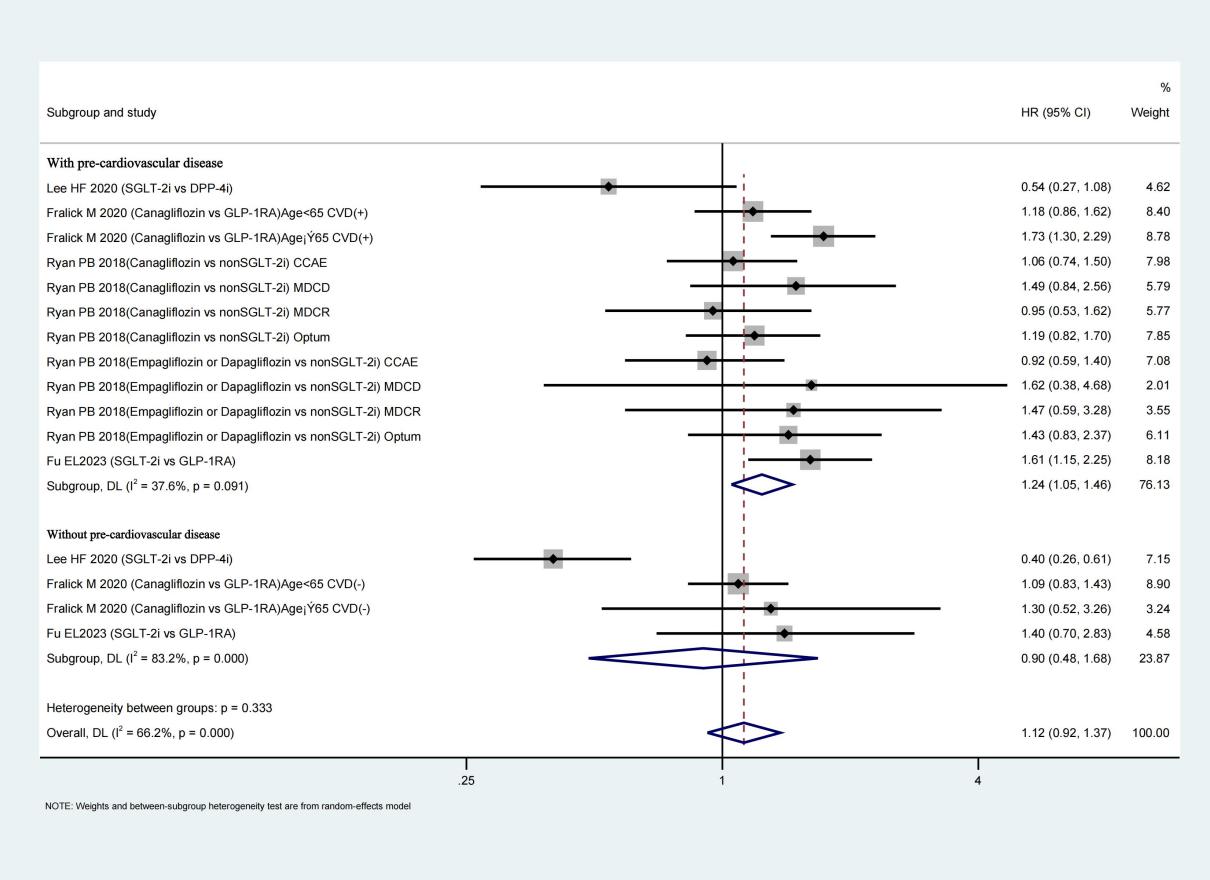


Legend: SGLT-2i = sodium-glucose cotransporter 2 inhibitors, GLP-1RA = glucagon-like peptide-1 receptor agonist, DPP-4i = dipeptidyl peptidase 4 inhibitors, CVD = cardiovascular disease

**5.7 Forest plot of SGLT-2i vs DPP-4i/GLP-1RA for urinary tract infections**


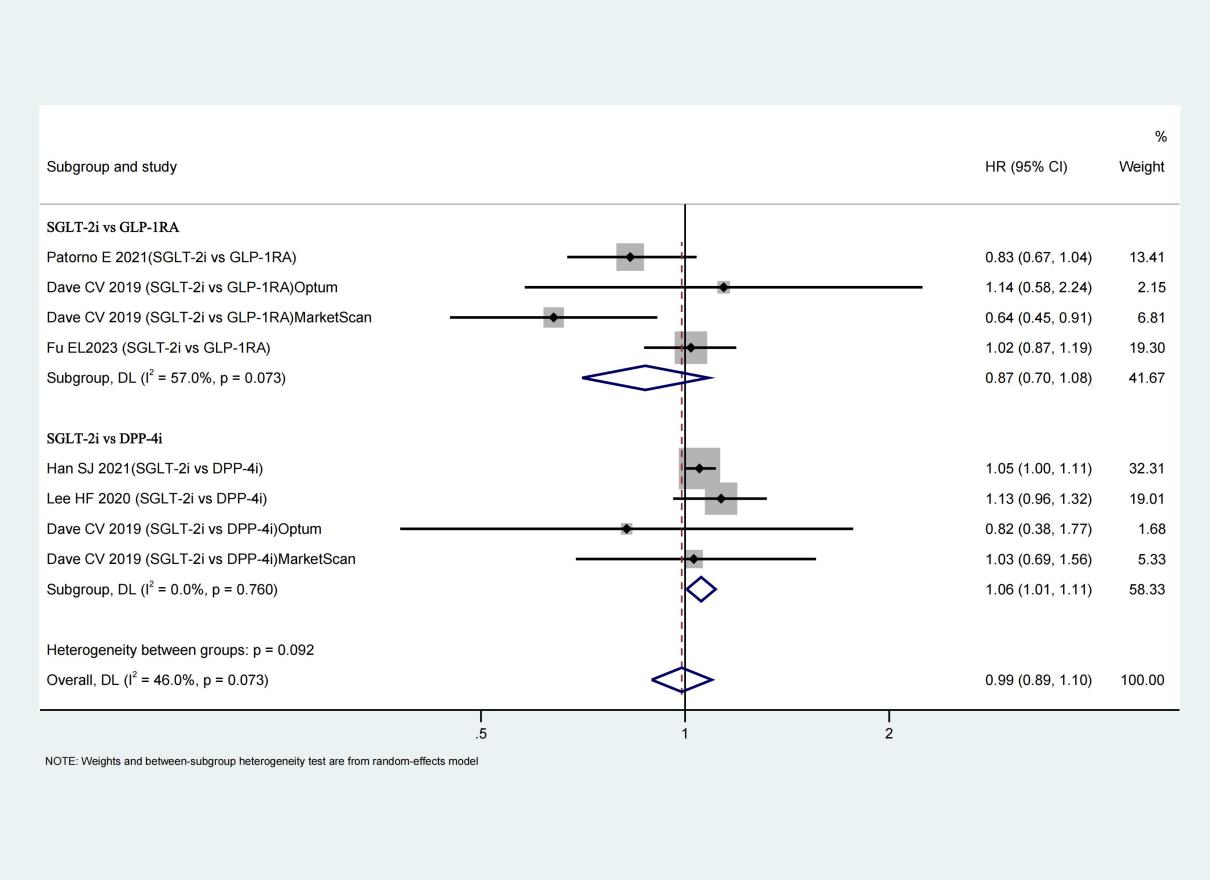


Legend: SGLT-2i = sodium-glucose cotransporter 2 inhibitors, GLP-1RA = glucagon-like peptide-1 receptor agonist, DPP-4i = dipeptidyl peptidase 4 inhibitors

**5.8 Forest plot of SGLT-2i vs DPP-4i/GLP-1RA for genital tract infections**


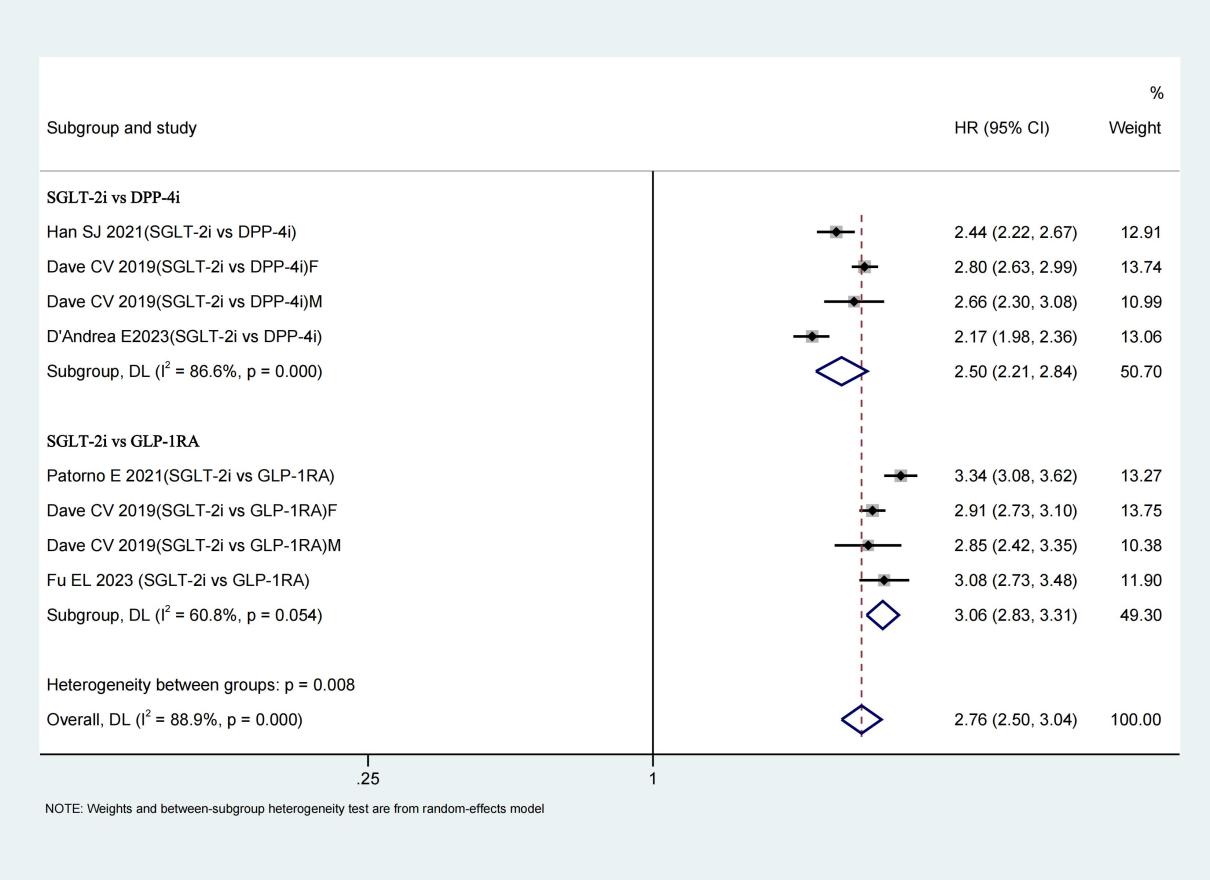


Legend: SGLT-2i = sodium-glucose cotransporter 2 inhibitors, GLP-1RA = glucagon-like peptide-1 receptor agonist, DPP-4i = dipeptidyl peptidase 4 inhibitors

**5.9 Forest plot of genital tract infections in female and male patients**


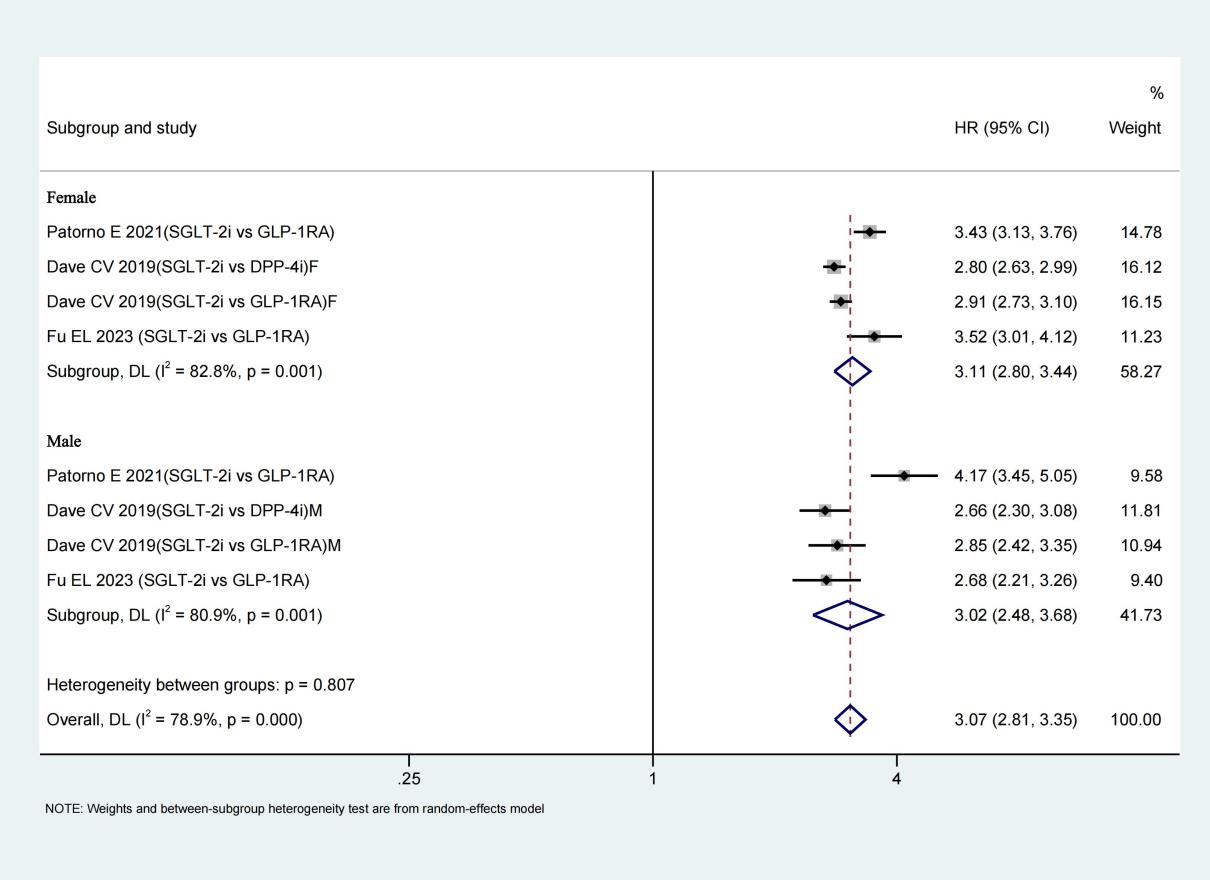


Legend: SGLT-2i = sodium-glucose cotransporter 2 inhibitors, GLP-1RA = glucagon-like peptide-1 receptor agonist, DPP-4i = dipeptidyl peptidase 4 inhibitors

**5.10 Forest plot of SGLT-2i vs DPP-4i/GLP-1RA for bone fracture**


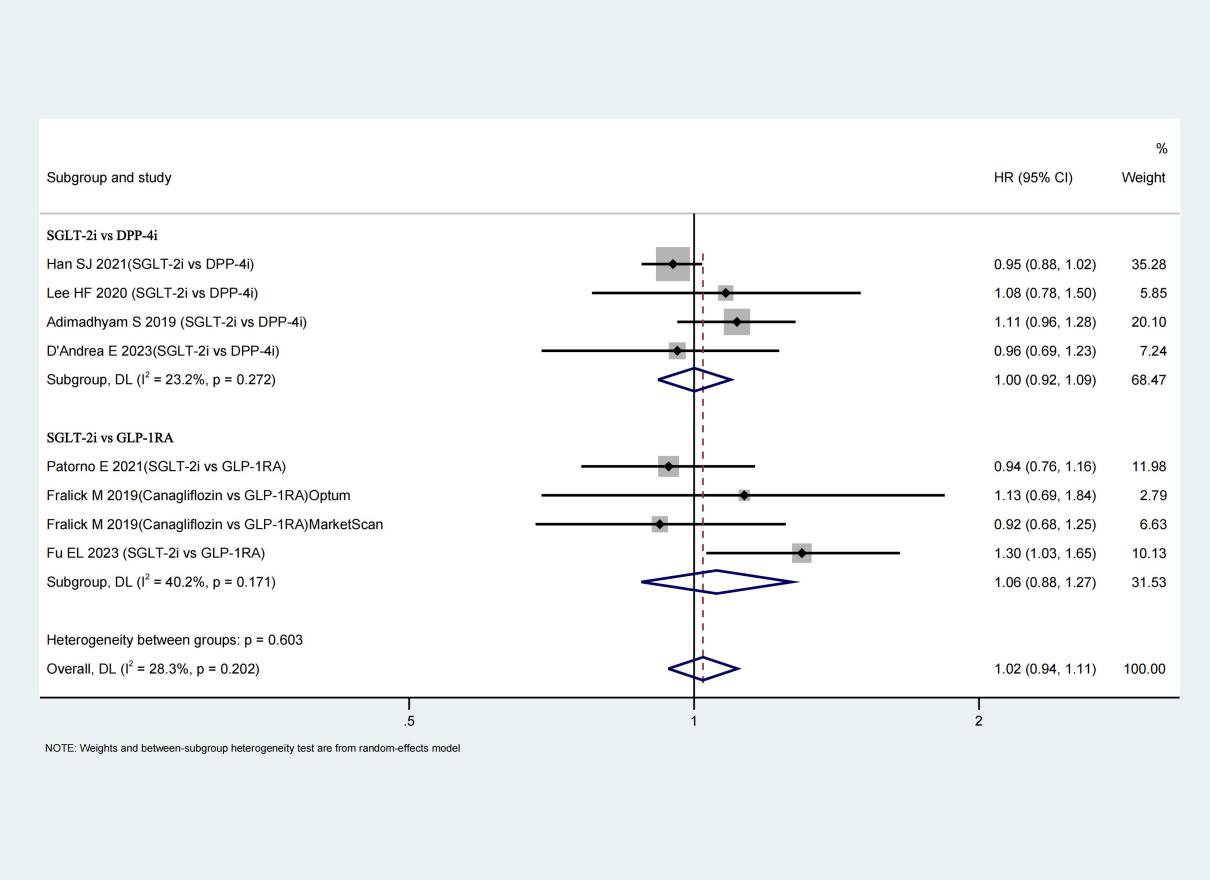


**Appendix 6: Results of meta regression analysis (*P***^†^**)**

| outcomes^‡^ | intervention | region | Year | Female | Follow-up time(m) | sample size |
| --- | --- | --- | --- | --- | --- | --- |
| DKA | 0.66 | 0.07 | 0.67 | 0.36 | 0.22 | 0.13 |
| LLA | 0.82 | 0.30 | 0.44 | 0.43 | 0.19 | 0.78 |
| UTI | 0.51 | 0.90 | 0.80 | 0.80 | 0.80 | 0.98 |
| GTI | 0.41 | 0.11 | 0.08 | 0.12 | 0.15 | 0.07 |
| bone fracture | 0.55 | 0.16 | 1.00 | 0.26 | 0.58 | 0.36 |
| hypoglycemia | 0.06 | 0.05 | 0.06 | 0.08 | 0.08 | 0.51 |

Legend:

† P <0.05 may be the factors affecting the heterogeneity of the pooled outcome

‡ DKA = diabetic ketoacidosis, LLA= lower limb amputation, UTI = urinary tract infection, GTI = genital tract infection.

**Appendix 7: Results of sensitive analysis**

**7.1** **Sensitive analysis of diabetic ketoacidosis**

**7.2 Sensitive analysis of lower limb amputations**

**7.3 Sensitive analysis of urinary tract infections**

**7.4 Sensitive analysis of genital tract infections**

**7.5 Sensitive analysis of bone fracture**

**7.6 Sensitive analysis of hypoglycemia**

**Appendix 8: Confunnel plots**

**8.1 Confunnel plots for diabetic ketoacidosis**


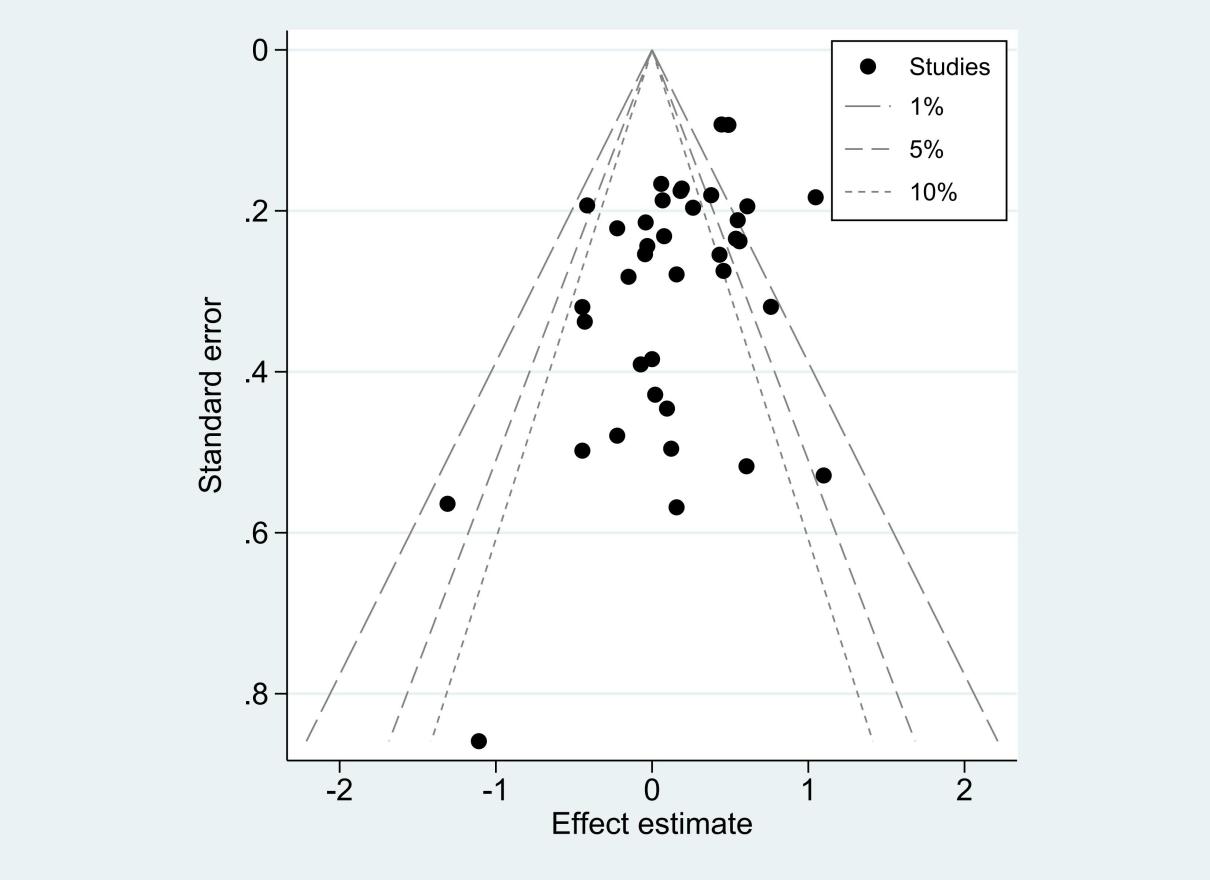


**8.2 Confunnel plots for lower limb amputations**

**
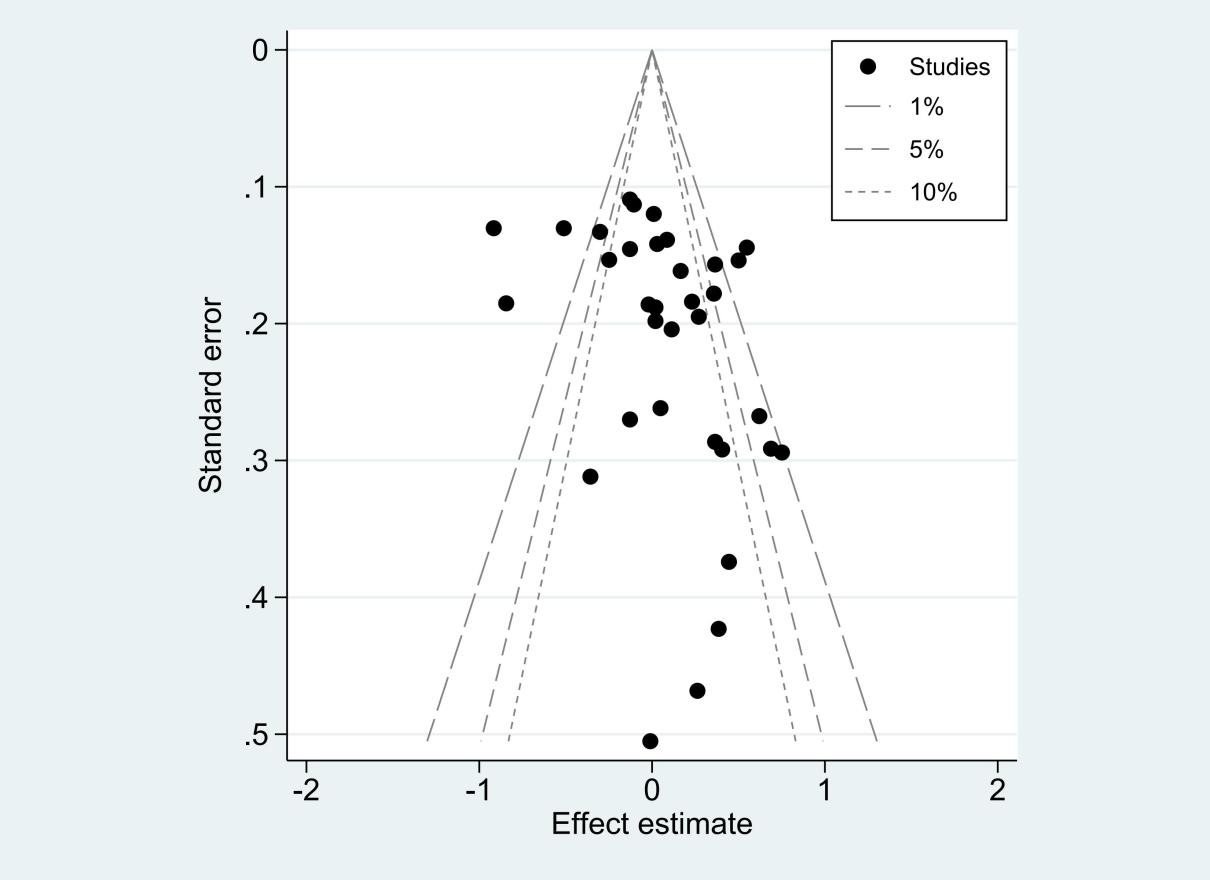
**

**8.3 Confunnel plots for urinary tract infections**

**
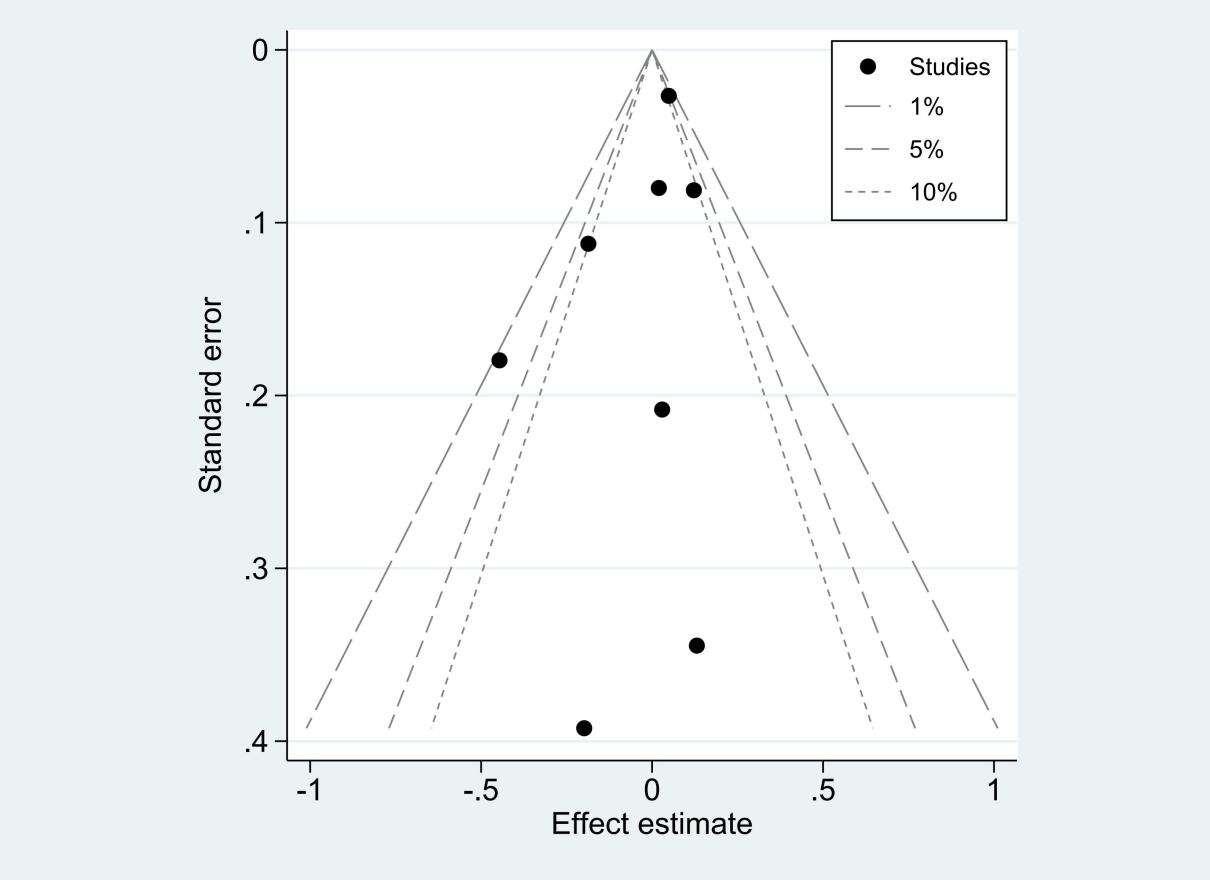
**

**8.4 Confunnel plots for genital tract infections**

**
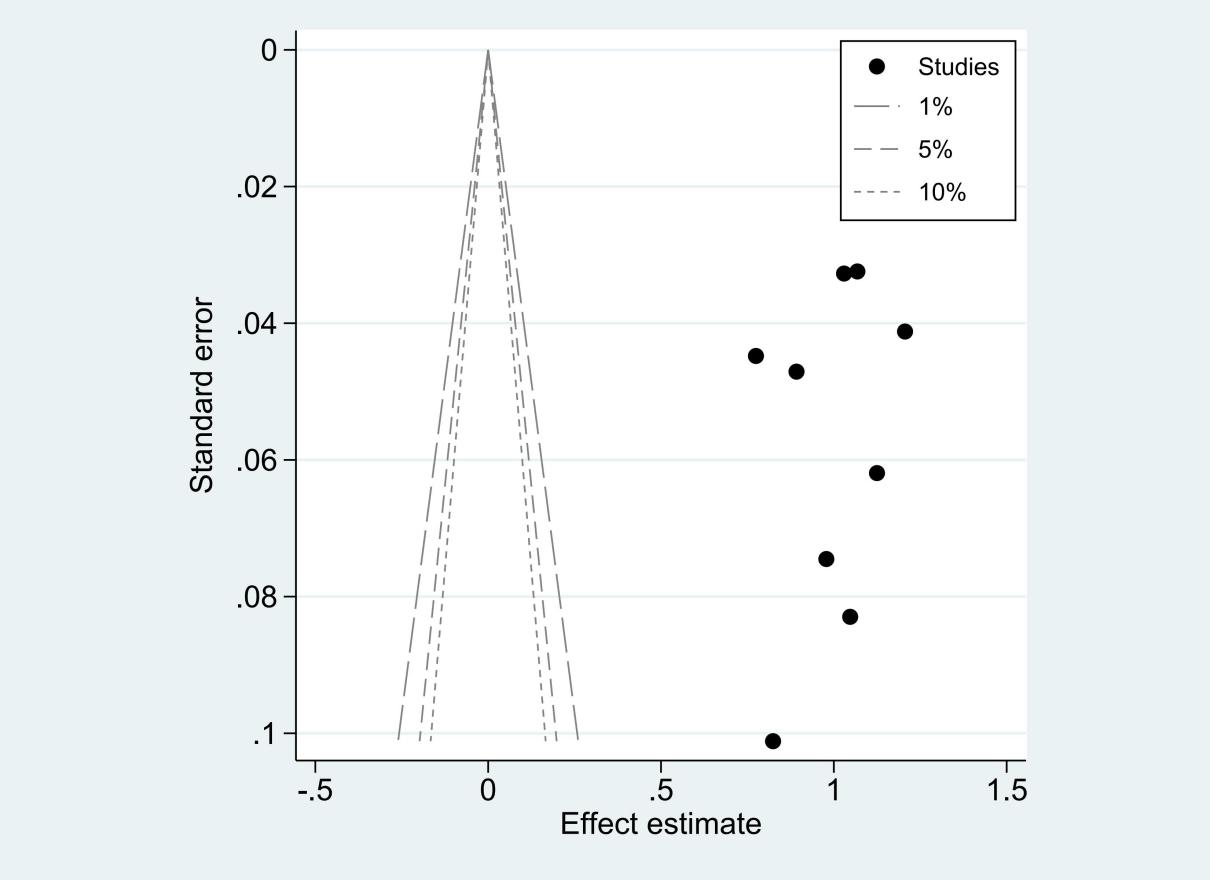
**

**8.5 Confunnel plots for bone fracture**

**
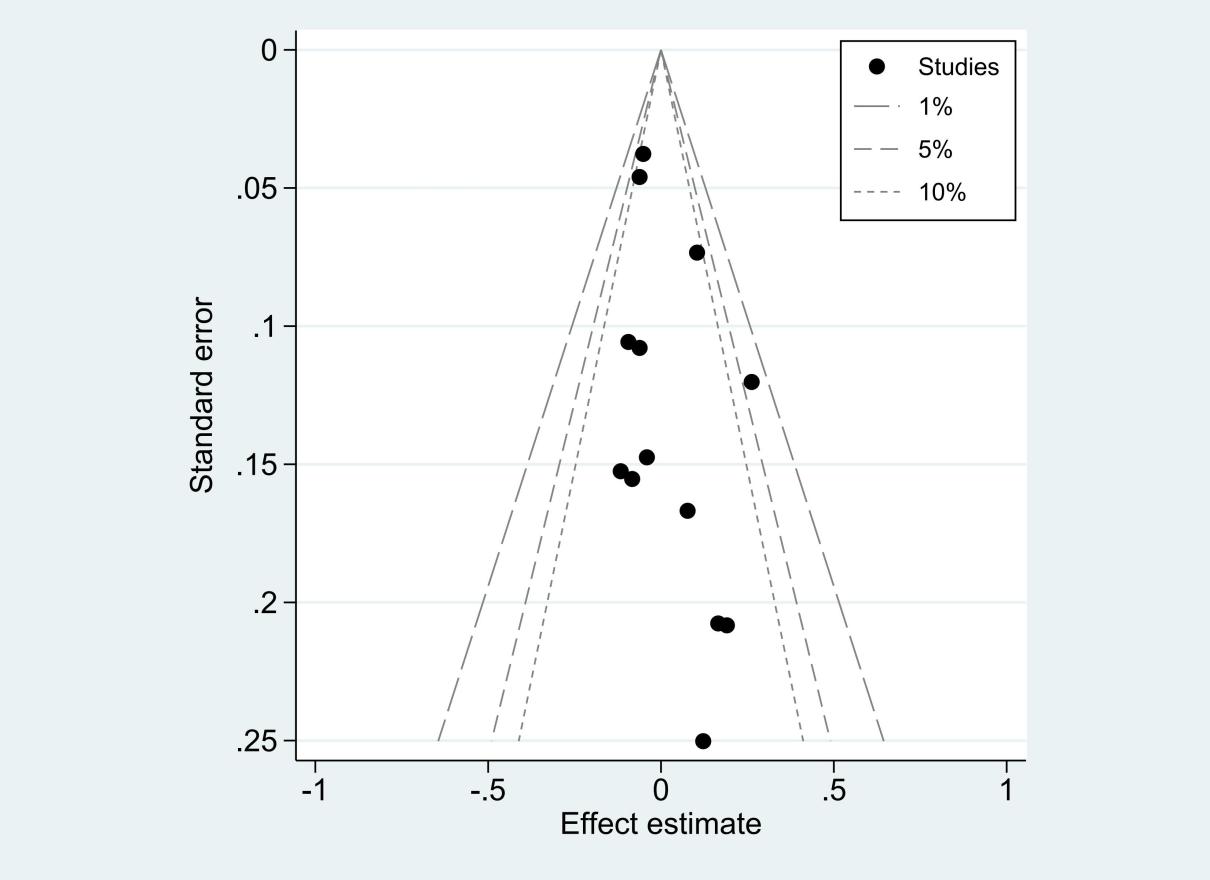
**

**8.6 Confunnel plots for [hypoglycemia](C:/Users/Administrator/AppData/Local/youdao/dict/Application/8.9.6.0/resultui/html/index.html#/javascript:;)**

**
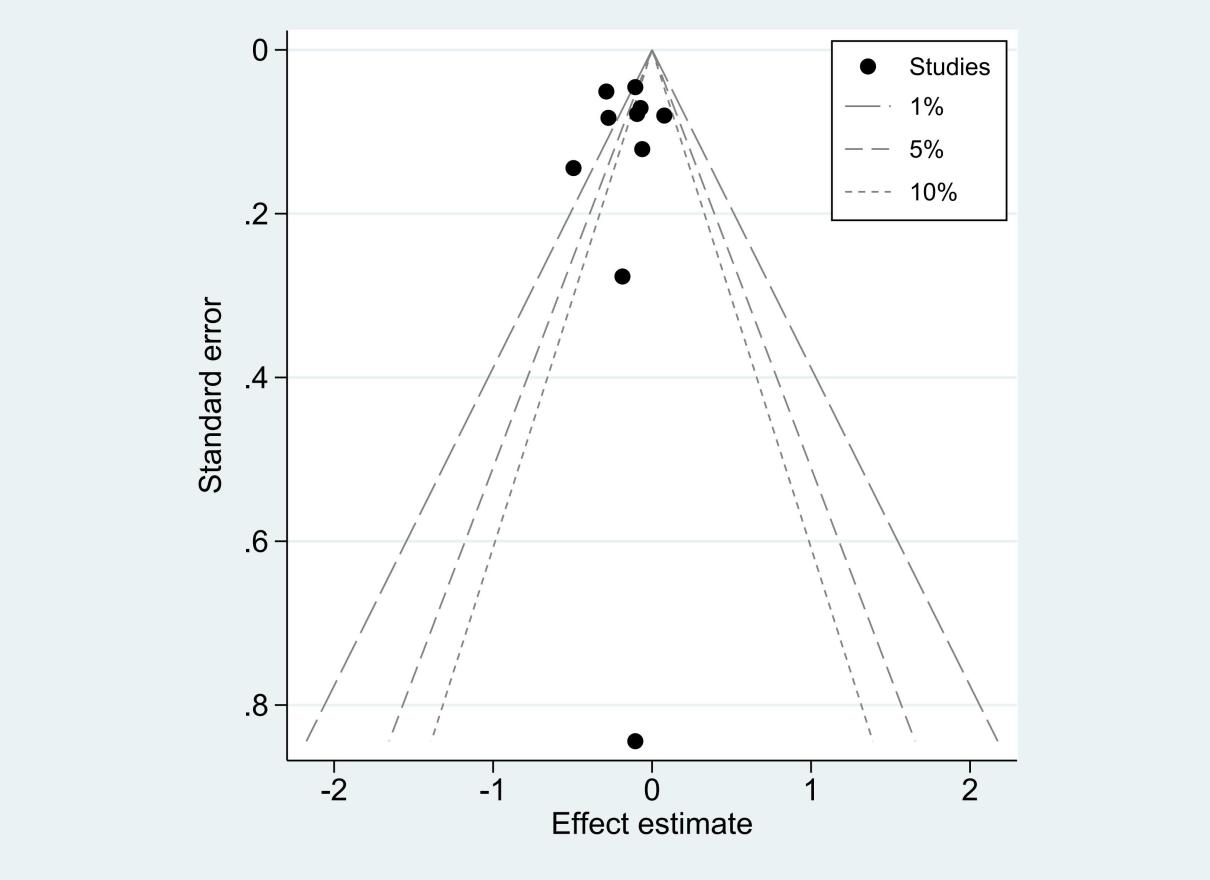
**

**Appendix 9: Results of subgroup analysis by study region**

| outcomes^†^ | region | Studies included | I^2^ | HR | 95% CI | P |
| --- | --- | --- | --- | --- | --- | --- |
| DKA | USA | 9 | 53.0 | 1.16 | 1.02,1.31 | 0.02 |
|  | Canada | 2 | 62.7 | 2.30 | 1.50,3.53 | ﹤0.01^**^ |
|  | Nordic | 2 | 48.0 | 1.56 | 0.76,3.20 | 0.22 |
| LLA | USA | 12 | 83.9 | 1.12 | 0.92,1.35 | 0.26 |
|  | Nordic | 2 | 0.00 | 1.03 | 0.91,1.16 | 0.63 |

Legend:

† DKA = diabetic ketoacidosis, LLA= lower limb amputation
